# Encoded diffractive optics for full-spectrum computational imaging

**DOI:** 10.1038/srep33543

**Published:** 2016-09-16

**Authors:** Felix Heide, Qiang Fu, Yifan Peng, Wolfgang Heidrich

**Affiliations:** 1 King Abdullah University of Science and Technology, Thuwal, 23955-6900, Kingdom of Saudi Arabia; 2 The University of British Columbia, Vancouver, BC V6T1Z4, Canada

## Abstract

Diffractive optical elements can be realized as ultra-thin plates that offer significantly reduced footprint and weight compared to refractive elements. However, such elements introduce severe chromatic aberrations and are not variable, unless used in combination with other elements in a larger, reconfigurable optical system. We introduce numerically optimized *encoded* phase masks in which different optical parameters such as focus or zoom can be accessed through changes in the mechanical alignment of a ultra-thin stack of two or more masks. Our encoded diffractive designs are combined with a new computational approach for self-calibrating imaging (blind deconvolution) that can restore high-quality images several orders of magnitude faster than the state of the art without pre-calibration of the optical system. This co-design of optics and computation enables tunable, full-spectrum imaging using thin diffractive optics.

In conventional imaging systems great efforts have been made to combat aberrations of all kinds by designing an increasingly large number of refractive or reflective lenses^1^,^2^. The number of elements in a commercial digital single-lens reflex (DSLR) camera, for example, can easily reach more than a dozen. As another example, groups of doublets or triplets are necessary in high quality microscopes to achieve diffraction-limited performance. In addition, more complicated imaging systems, e.g. zoom lenses, frequently require additional longitudinally moving groups to change the focal lengths in a specific range^3^,^4^. Furthermore, special glass with extraordinary dispersion properties is incorporated into many designs to minimize primary and higher order chromatic aberrations. Consequently, conventional imaging systems are, in most cases, bulky, heavy, inflexible, and costly.

A promising avenue for reducing the size and complexity of conventional lenses is to replace complex components with simpler and smaller elements in combination with computational techniques. Instead of eliminating aberrations optically, the aberrations in a simplified optical system can also be designed for easy computational removal, for example by favoring point spread functions (PSFs) that preserve high spatial frequencies. Diffractive optical elements (DOEs) in particular are an interesting replacement for complex imaging systems. DOEs can be fabricated on ultra-thin plates and are very flexible in modulating light, thus delivering greater freedom in design parameters. One drawback, however, is that they introduce strong chromatic aberrations^5^ which limit their application in colour imaging. Computational imaging techniques have proven to be able to digitally correct optical aberrations^6^,^7^ including recent work by Heide *et al.*^8^ which proposed a statistical prior for reducing chromatic aberrations from single element optical systems.

In recent years there have been a large number of computational imagers that employ a co-design of refractive or diffractive optics and computation. Examples include cubic phase plates for extended depth of field imaging^9^, DOEs producing double-helix PSFs^10^ for single-molecule microscopy beyond the diffraction limit, phase microscopy^11^, coded amplitude masks instead of lenses^12^, and anti-symmetric gratings integrated with a complementary metal-oxide-semiconductor (CMOS) sensor to produce an ultra-miniature lens-less imager PicoCam^13^,^14^,^15^. Although these approaches have demonstrated the possibility to develop imaging systems with reduced optical complexity by shifting the burden to computational reconstruction, the resulting systems exhibit little flexibility, such that refocusing or zooming are either not supported at all, or require tedious re-calibration. Additional shortcomings of existing systems are poor image quality and lack of colour imaging for diffractive designs, as well as high computational cost.

In this work, we present a high resolution, broadband (i.e. colour) diffractive imaging technique that jointly exploits computationally optimized diffractive optics and computational image reconstruction. The overview of our system is shown in Fig. 1.

We computationally design stacks of two or more DOEs in order to encode arbitrary lenses for different geometric configurations of DOEs. The relative positioning of these DOEs (i.e. different rotations or translations) encodes different optical designs such as a variety of focal lengths or parameters of a cubic phase plate. Our approach is a generalization of the work^16^,^17^ which presented an analytical phase pattern for two rotating DOEs to change focus. We achieve general encodings of lenses by formulating the design as a numerical optimization problem. This optimization problem turns out to be a complex matrix factorization problem. A novel, efficient optimization method allows us to solve this problem and thus design two or more DOEs such that, when arranged in different geometric configurations (e.g. rotations or translations), complex transmission functions corresponding to different target lenses can be generated. Other analytic designs have been described and analyzed in the work^18^,^19^,^20^,^21^. Note that all of these approaches propose analytic solutions for selected target designs, while our method numerically optimizes for phase patterns given arbitrary target designs.

Aberrations are deliberately tolerated in the images captured using our optical designs and removed by a new self-calibrating computational reconstruction. This blind image reconstruction method jointly estimates the underlying scene-dependent, spatially-varying wavelength dependent point spread functions (PSFs) and the latent sharp image. Our method adaptively self-calibrates the PSFs from the image itself as well as reconstructs sharp images without any physical calibration of the imaging system for all possible geometric configurations. Exploiting statistical cross-channel correlation as part of our joint reconstruction, major issues of current state-of-the-art blind PSF estimation methods can be eliminated. We show that our method can restore high quality images exceeding the image quality of previous fully calibrated (i.e. non-blind) approaches, while being 5 orders of magnitude faster than the state of the art.

We experimentally show three applications of our flexible encoded diffractive lens: static colour imaging with a single diffractive interface, rotational refocusing and rotation-only zooming. In simulation, we furthermore demonstrate additional applications including shift-only focus changing, adjustable cubic phase plates and time-multiplexed diffractive lenses. The proposed technique reveals its versatility and potential for achieving lighter, more compact, more flexible, and more powerful imaging systems.

## Results

Our encoded diffractive imaging system consists of a numerically optimized optical design and self-calibrated reconstruction as explained above. In the following, the computational design of the encoded optics is discussed first. After that, the reconstruction approach is described, which takes the corrupted measurements as the input. In these two parts we show the design results, reconstruction process and experimental results in different imaging scenarios.

### Encoded diffractive lens

Consider a rotational encoding of different phase function designs where each design is implemented by a different rotation angle of two phase gratings. We describe the complex target transmission function as the product of two transmission functions of the individual DOEs. For special transmission functions such as Fresnel lenses, approximations can be found analytically^16^,^17^, but these methods do not generalize to arbitrary target transmission functions, and the accuracy of the approximation is limited. We instead reformulate the design problem as a matrix factorization problem, whereby a general target transmission function is represented as a matrix that can encode all possible geometric alignments between the DOEs since it contains an entry for every combination of pixels on the first and second DOE. A stack of two static DOEs, can produce a Rank-1 approximation of this complex matrix, which can be found through complex matrix factorization (see Methods and Supplementary Methods). Note, however, that mutual rotation cannot “perfectly” encode optical elements with non-rotational symmetric targets in a continuous angular range.

The described principle can be generalized in several ways: stacking more than two diffractive masks results in a tensor factorization problem rather than a matrix factorization problem. If one or more of the DOEs are replaced with a dynamic phase modulator that allows for temporally multiplexing different diffractive patterns, then the rank is increased to the number of different patterns displayed during a single exposure. In this setting super-resolved features and higher bit-depth than for a single phase modulator might be achieved^22^.

Any target phase functions can be encoded including Fresnel lenses, axicons^23^, and cubic phase plates^24^. Figure 2 shows the phase profiles of both the target lenses and the approximated results using our factorization methods. Varifocal lenses can be achieved by encoding focal lengths into mutual rotation of two layers using Rank-1 matrix factorization (Fig. 2(a)) for two static elements. Similarly, focal lengths (Fig. 2(b)) or cubic phase functions (Fig. 2(c)) can be approximated by relative translation of two layers using Rank-1 factorization. In the time-multiplexing case, higher rank factorization can be used. A focal length encoding example of this kind is also shown in Fig. 2(d) using Rank-4 factorization.

For experimental verification, we designed and built four rotationally encoded lenses. All the lenses are designed at wavelength *λ* = 550 *nm* on 0.5 *mm* thick Fused Silica substrates with an aperture diameter of 8 *mm*. The focal length ranges are [100 *mm*, 200 *mm*], [100 *mm*, 300 *mm*], 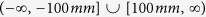, and 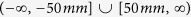 respectively.

### Reconstruction algorithm

Raw images captured by our encoded lens suffer from inherent chromatic aberrations introduced by the wavelength-dependency property of DOEs. Therefore, computationally reconstructing images without aberrations is a key part in our imaging system. We devise a fast blind deconvolution process that *jointly* performs PSF self-calibration and deconvolution.

**Figure 1:**
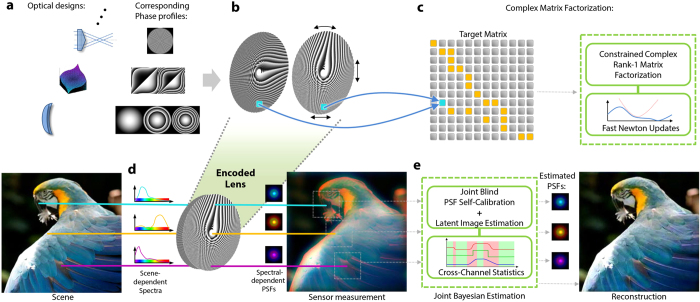
Overview of the imaging system. We computationally design stacks of DOEs (**b**) that can encode arbitrary lenses for different geometric configurations, such as relative shift or rotation. Examples include focus-tunable lenses, tunable cubic phase-plates, and axicons shown in (**a**) with their corresponding phase transmission functions. Given a target phase, we design two or more phase plates using complex matrix factorization (**c**). If more than two phase plates are used even complex optics like zoom-systems can be designed. A novel computational approach enables broadband imaging for our encoded lenses (**d**,**e**). When a scene is imaged with our diffractive encoded lens, points with different spectral distributions result in significantly different PSFs. Our reconstruction algorithms (**e**) jointly self-calibrates the spatially-varying, scene-dependent PSFs and recovers the latent image exploiting cross-channel statistics. Aberrations in the reconstruction are effectively removed (actual reconstruction shown here).

**Figure 2:**
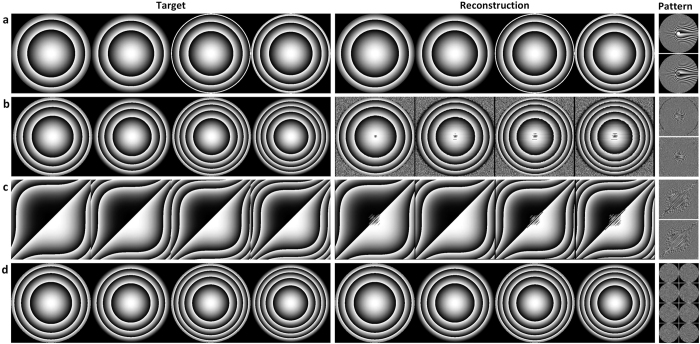
Encoding examples of the optimization for different target phase functions. (**a**) Focal lengths are encoded into mutual rotation of two layers using Rank-1 complex matrix factorization. (**b**) Focal lengths are encoded into translation of two layers using Rank-1 complex matrix factorization. (**c**) Cubic phase functions are encoded into translation of two layers using Rank-1 complex matrix factorization. (**d**) Focal lengths are encoded into time-multiplexing using Rank-4 complex matrix factorization. From left to right: four target phase functions, reconstructed phase function and their respective optimized phase patterns.

Note that, in general, different spectral distributions of objects and illumination cause spatially varying PSFs for a diffractive lens when measured on a colour sensor. This effect is shown in Fig. 1. Consider the three points with different spectra shown in Fig. 1(d), the response of the lens and sensor lead to differently coloured PSFs (cyan, yellow, and magenta in Fig. 1(d)) in different sub-regions of the image. Material and illumination properties, on the other hand, are spatially low-frequency which result in a PSF that is spatially invariant in a local neighbourhood.

To handle such spatially varying PSFs our reconstruction method exploits cross-channel correlation as a statistical prior. In particular, the empirical distribution of gradient differences between two spectral bands turns out to be heavy-tailed (see Supplementary Fig. 3). Intuitively this means gradients of two colour channels are very similar in most areas except for occasional pronounced changes in chroma and luma (e.g. object or material boundaries). A nice property of the encoded diffractive lens is that, when taking an RGB image, at least one channel can be focused well while the other two channels are blurry. This structure is directly exploited by the cross-channel prior. Note that our formulation model differs from that previously proposed^8^ which only assumes chroma changes to be sparse. This assumption leads to severe instabilities of the prior for low intensities.

Using this modified cross-channel prior we jointly estimate the spatially varying PSFs and the latent sharp image. Hence, the reconstruction process is self-calibrated and adaptive to the scene. Compared to other common methods which require tedious and complicated physical calibration, our method avoids these steps for all possible geometric configurations and is therefore more convenient and robust.

It turns out that adding the prior in the blind deconvolution converges to a significantly better optimum in drastically less computational time than competing methods. We are able to reconstruct images 5 orders of magnitude faster than state of the art with drastically improved quality. See Methods and Supplementary Methods for an in depth discussion of our reconstruction approach including comparisons.

### Experimental setup

**Figure 3:**
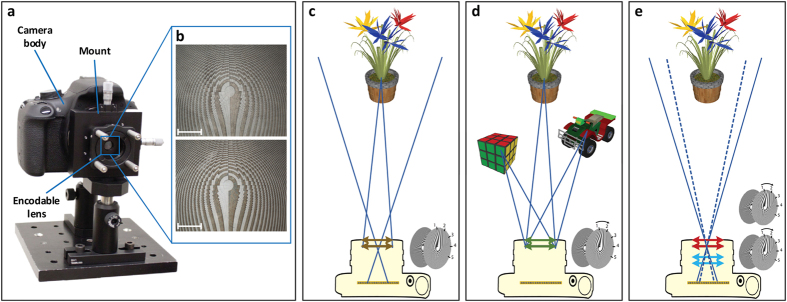
Experimental set-up. (**a**) Our encoded imaging system consists of a encoded lens, mechanical mounts and a camera body. The encoded lens is mounted on a 3-degree-of-freedom adjustment cage system. A Canon EOS Rebel T5 camera body is connected to the mounting system by a lens tube to prevent stray light. (**b**) The central areas of the two component DOEs in the design with focal length range 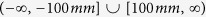 are observed on a microscope Nikon Eclipse L200N with a 5× objective. The scale bar is 500 *μm*. (**c**) Set-up of the static broadband imaging system. Without movement, our system is able to image the scene as a conventional lens. Our computational approach reconstructs high quality output images from the corrupted measurements. (**d**) Set-up of the rotational refocusing system. With only mutual rotation, our system is able to refocus on different objects within an extremely large depth of field. (**e**) Set-up of the rotation-only zooming system. Employing two encoded lenses, our system is able to zoom in or out with only rotation required to keep the objects sharp. The illustrations in this figure were generated using Sketchup^26^.

Our encoded diffractive lens system is shown in Fig. 3(a). The refractive lens on a Canon EOS Rebel T5 DSLR is replaced by a group of encoded diffractive lenses consisting of two DOEs stacked face-to-face. The encoded lens is placed at the flange focal distance (44 mm) of the camera which contains a APS-C sensor (22.3 mm × 14.9 mm). The phase patterns are fabricated using photo-lithography (see Methods and Supplementary Methods for details) on 4” Fused Silica wafers. Figure 3(b) shows the 5× microscopic images of the central areas of both DOEs. When the two elements are rotated relative to each other, the equivalent focal length varies as a function of the rotation angle. In order to achieve precise alignment (tilt and decenter) between the two DOEs, we first mount the two elements on two cage rotation mounts (CRM1/M, Thorlabs) respectively. The cage systems are then mounted in an XY translator with micrometer drivers (ST1XY-S/M, Thorlabs). This three degree of freedom mounting system guarantees accurate alignment as well as smooth 360° rotation. The mounted lens is then connected to the camera body with a lens tube.

Three applications of the encoded diffractive lens are illustrated in Fig. 3(c–e). First, the static configuration of the system is a traditional diffractive lens at a specific focal length (Fig. 3(c)). Second, when the distance between the encoded lens and the sensor is fixed, the lens can focus on objects at different distances (Fig. 3(d)) if one element is rotated with respect to the other. Third, a rotation-only zoom lens can be achieved by two encoded lenses separated by a short distance. When the two groups are rotated at specific angles respectively, the image can be zoomed in or out while remaining sharp. No longitudinal movement is involved leaving the overall length of the system unchanged. In all cases, a high quality image is reconstructed by the proposed joint blind PSF estimation and deconvolution approach.

### Static colour imaging

**Figure 4:**
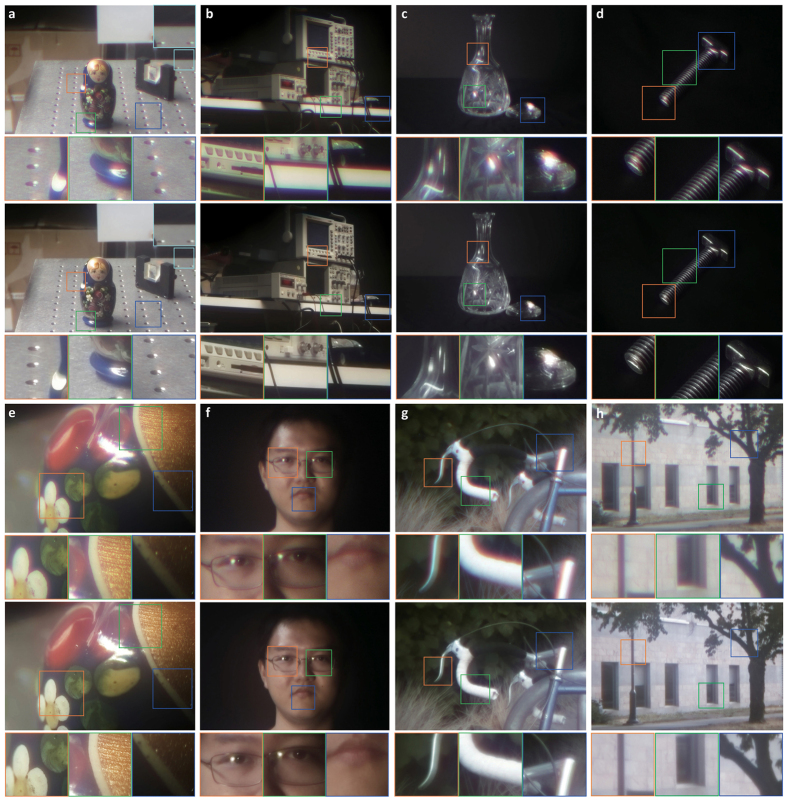
Results of static colour imaging with a single encoded lenses. For each individual result, both the blurry observation (top) and reconstruction (bottom) are shown for indoor and outdoor natural scenes. (**a**,**b**) Indoor scenes under fluorescent lamp illumination. (**c**) Transparent objects with reflective surfaces. (**d**,**e**) Small objects imaged in macro mode. (**f**) Human face. (**g**,**h**) Outdoor scenes under sunlight illumination. Insets in each result highlight the details before and after computational reconstruction. Chromatic aberrations are completely removed in all scenarios. The focal length range of the encoded lens for experimental results shown here is [100 *mm*, 300 *mm*].

When the two DOEs are stationary with respect to each other, the equivalent phase is a diffractive lens with a specific focal length. The focal length can range from a few millimetres to nearly infinity. In all cases, we have focused our lens for the green channel, that is, the central part of the green channel’s spectral response. We show several results for different scenes captured with different focal length settings in Fig. 4 to demonstrate the flexibility of encoding focal lengths into relative rotation.

Figure 4 shows results of our encoded lens in static colour imaging. The indoor illumination is a fluorescent lamp. Although strong colour fringes occur at edges (Fig. 4(a,b)), especially for white objects, these chromatic aberrations introduced by the diffractive lens have been completely removed in the reconstructed image (see the corresponding insets for highlights). The algorithm is also robust to transparent materials with reflective surfaces (Fig. 4(c)). In addition to imaging similarly to a conventional lens, our encoded diffractive lens can also perform in macro mode to image very small objects (Fig. 4(d,e)). For natural scenes such as human faces (Fig. 4(f)), the algorithm is also able to reconstruct a high fidelity image. Outdoor scenes under sunlight illumination are shown in Fig. 4(g,h). Note that, although the aberrations are depth and wavelength dependent, both the foreground and background objects can recovered simultaneously to extend the depth of field.

**Figure 5:**
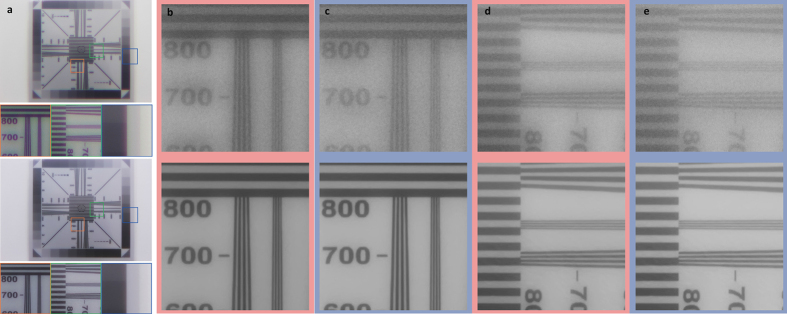
Resolution chart captures with a single encodable lenses. In (**a**) the blurred measurement (top) and the corresponding reconstruction (bottom) are shown along with a few extracted patches. The red and blue colour channels of these patches are shown in (**b**–**e**), the green channel was focused. The result (**b**) shows the red channel with the blurry capture (top) and the reconstruction (bottom). (**c**) shows the blue channel for the same patch. (**d**,**e**) shows the same channels for a different patch. We can see that for both blurred channels our reconstructions have a drastic increase in resolution. The focal length range of the encoded lens for experimental results shown here is [100 *mm*, 200 *mm*].

The resolution chart capture in Fig. 5 shows that resolution is increased while eliminating chromatic aberrations. Having focused our lens again for the green channel, we can see that severe aberrations occur for the red and blue channel in (b–e). The proposed approach successfully eliminates these aberrations and recovers lost resolution. Especially the high resolution TV lines are reconstructed accurately for all patches shown in Fig. 5. Note that no deconvolution artefacts (e.g. ringing) are introduced. Our method is robust to noise and removes the strong noise in the patches from Fig. 5 which usually causes deconvolution artefacts. See the Supplement Methods for a detailed discussion of how our approach can handle Poisson-distributed noise.

### Rotational refocusing

Since the focal lengths change along with rotation angles, our encoded lens can perform refocusing with only mutual rotation while the distance between the lens and the sensor remains fixed. This is particularly useful when space is limited and longitudinal movement of the lens is prohibited.

**Figure 6:**
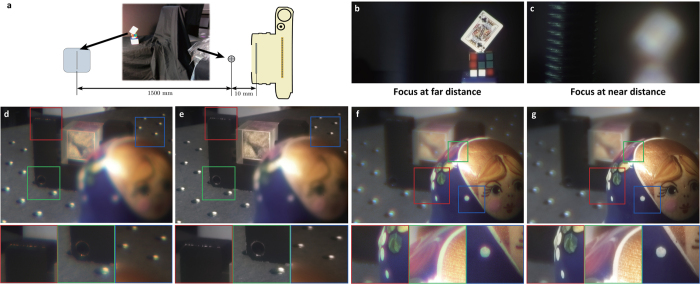
Results of refocusing with relative rotation between two DOEs. (**a**) A screw is located 10 *mm* in front of the lens and a poker card on a magic cube is located 1500 *mm* further away. (**b**) When the lens is focused on the rear poker card, the front screw is hardly seen due to the strong out of focus. (**c**) When the lens is focused on the screw, the poker card is severely blurred. (**d**) Captured image when the lens is focused on the rear beam-splitter cage. (**e**) Reconstructed image of (**d**). (**f**) Captured image when the lens is focused on the foreground doll. (**g**) Reconstructed image of (**f**). Insets of the results from (**d**,**e**) indicate that our algorithm is robust to both saturated and dark areas in the same scene. Subfigure (**b**,**c**) had to be cropped for copyright reasons. The focal length range of the encoded lens for experimental results shown here is 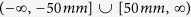.

Figure 6(a) illustrates a refocusing setup with an extremely large depth of field. A very small screw is located 10 *mm* in front of the lens and a poker card on a magic cube is located 1500 *mm* further away. Our system can easily focus on both the far objects (Fig. 6(b)) and near objects (Fig. 6(c)) through rotation. When the poker card is focused, the screw is hardly seen due to strong out of focus blur. Similarly, when the screw is in focus the poker card is severely blurred. An example of a more complicated scene is shown in Fig. 6(d). The lens is focused on the far beam-splitter cage and the near Matryoshka doll is out of focus while in Fig. 6(f), the near doll is focused instead of the far cage. The sharp images are shown in Fig. 6(e,g) respectively. Insets show that our algorithm can restore details very well (e.g. the screw holes on the table). Note that the saturated area on the doll and the very dark area on the corner of the black cage indicate that our algorithm is also robust to different exposure levels.

### Rotation-only zooming

Conventional zoom lens requires two types of motion to change the image magnification while maintaining focus. First is the longitudinal moving of an inner group of lenses within the entire lens to change the focal length, and the second is longitudinal moving of the whole lens to refocus. Therefore, zoom lenses are usually bulky, heavy and require precise mechanical components to maintain lens alignment while moving^4^.

Benefiting from the focal length encoding capability of our encoded lens, we are able to realize rotation-only zooming without longitudinal movement. As shown in Fig. 3(e), our zoom lens consists of two encoded lenses separated by a fixed distance. This is a two-component system in which the object distance, image distance and separation between the two lenses are all fixed. There exists a single pair of focal lengths that correspond to a specific magnification^3^. Therefore, to achieve a change in magnification (i.e. zooming) the focal lengths *f*_1_ and *f*_2_ must change according to the relationship


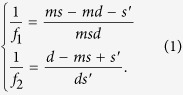


where the object distance *s*, image distance *s*′ and spacing *d* between the two groups of DOEs are all fixed.

**Figure 7:**
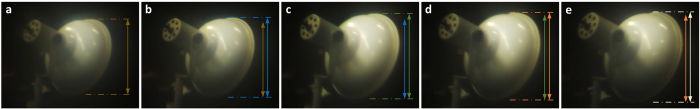
Results of rotation-only zooming with two groups of encoded lenses. A sequence of images of the same lamp from (**a**–**e**) indicate the magnification change. Arrows in the same colour have the same size in all the images.

Our two-group encoded lens system is able to achieve rotation-only zooming by finding different sets of focal length parameters. All the adjustments involved are implemented by mutual rotation of the two DOEs in a single group. Figure 7 shows a set of reconstructed images captured at different magnifications. In each image auxiliary arrow marks indicate the magnification change compared to the previous image. Note that due to the Chromium (Cr) aperture of our DOEs the image quality is somewhat degraded compared to the static broadband results because of the inter-reflection between the two groups of DOEs.

## Discussion

The combination of computationally optimized diffractive optics and computational reconstruction opens up new territory for high quality broadband computational imaging.

In this work we have achieved several major advances in imaging technology. First, we demonstrate that pairs of diffractive optical elements can be computationally designed to encode a wide range of optical parameters into simple mechanical rotations and translations. Thin lenses of varying focal lengths, or cubic phase plates and axicons with different parameters can all be achieved with continuous rotational or translational changes in alignment. More complex optical systems such as zoom lenses can be assembled with two or more pairs of DOEs. These are initial examples of this design approach, which should, however, apply much more widely, thus paving the road for significantly more flexible future imaging systems.

Secondly, we show that, using computational imaging approaches, such encoded diffractive optical elements can be used for full-spectrum and colour imaging. We believe this is the first time high-fidelity colour imaging has been demonstrated using only diffractive optical components.

Finally, we make significant improvements on two salient problems with many computational imaging methods: calibration effort and computational expense. We demonstrate a fully self-calibrated imaging system, in which image restoration is achieved on individual images without additional calibration information. This method is also significantly faster than existing image restoration methods without sacrificing reconstruction quality.

Encoded diffractive optics generalize well to wavelengths outside the visible spectrum. For imaging in a narrow spectral band at any frequency, the only requirement is that diffractive optical elements can be fabricated for that wavelength. For broadband imaging, we additionally require that the image sensor has multiple spectral channels, one of which corresponds to the design wavelength of the diffractive optical elements. We believe that this property makes encoded diffractive optics particularly interesting in the UV or THz range, where refractive optics is either expensive or not available at all.

## Methods

### Encoded diffractive lens design

The design of encoded diffractive lens is a complex tensor factorization problem. The target transmission function **T** in polar coordinates (*r*, *ω*) can be described as the multiplication of two transmission functions **T**_1_ and **T**_2_


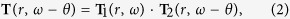


where we have encoded optical parameters into the relative rotation angle *θ* of the second element. Our target is to design a varifocal lens whose transmission function is


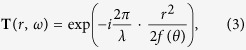


where *λ* is the design wavelength and *f(θ*) is the angle-dependent focal length. It is possible to rewrite the above equation as a complex matrix factorization problem by remapping polar coordinates (*r*, *ω*) to linear indices addressing the columns of two complex matrices **A** and **B**. We solve the following optimization problem


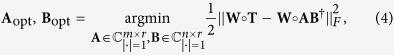


where **B**^†^ is the complex conjugate of **B**, ||·||_*F*_ denotes the Frobenius norm, and 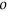 denotes the Hadamard product. A weighting matrix **W** is added to select only those matrices physically possible over each rotation angle. We refer the reader to Ho^25^ for a detailed introduction to weighted (non-negative) matrix factorization methods. For static DOEs, the **A**, **B** matrices are of rank 1, **AB**^†^ can also be interpreted as the inner product of two vectors that correspond to the mask pattern of each DOE. For dynamic patterns higher rank factorizations can be realized, as described at the beginning of the Results Section.

For our discussion, we assume the static Rank-1 setting, but we note that the same methods can be applied for higher rank factorizations. The bi-convex matrix factorization problem can be efficiently solved as a sequence of convex sub-problems using an alternating approach. In each sub-problem we diagonalize the weighting matrix and perform the outer vector product operation followed by vectorization. We can easily derive the gradient and the Hessian results in a diagonal matrix whose inversion using Newton’s method then becomes a point-wise division. By symmetry, both sub-problems can be solved very efficiently. See Supplementary Methods for an in-depth discussion.

### Self-calibrated blind deconvolution

The image reconstruction method jointly estimates the underlying spatially-varying PSFs and the latent sharp image. Having ensured that our encoded lens focuses well in at least one channel, we can solve for the PSFs by exploiting cross-channel correlation between the channels. In particular, we solve the following optimization problem:


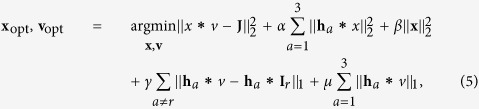


where **j** is the captured raw image, **v** is the unknown latent image, and **x** is the unknown PSF. The convolution of the latent image with the PSF is expressed as **x** ∗ **v** using the convolution operator ∗. Here **h**_*a*_ (*a* = 1, 2, 3) are first order spatial gradient filters for RGB channels, and **i**_*r*_ is a sharp image in reference channel *r*, and *α*, *β*, *γ* and *μ* are weights for the regularization terms. In the first row we have a classical 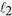 data term, a gradient prior and a low-energy term on the PSF **x** in the 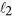 sense. The cross-channel prior on the latent image **v** in the second row of Eq. (5) penalizes the gradients between blur channels and the reference channel in the 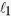 sense. A sparsity penalty on the gradients of images in all channels is further added. Please the Section 3 of the Supplemental Methods for a detailed discussion of all penalty terms from Eq. (5).

The bi-convex minimization problem in Eq. (5) can be solved via coordinate descent without any additional priors or optimization schedule tricks. We keep one of the two variables **x**, **v** fixed while minimizing the objective with respect to the other in an alternating fashion. This approach leads to the two sub-problems, **x**-step and **v**-step, both of which are much simpler to solve than the joint objective.

Minimizing the quadratic from the **x**-step is equivalent to solving a system of linear equations composed of structured matrices, all of which represent convolutions. This structure can be exploited by reformulating this linear equation system in the frequency domain and inverted efficiently by point-wise division (see Supplemental Methods for a derivation).

The **v**-step requires the solution of a deconvolution problem with known kernel **x**^*k*^. It involves a quadratic data term, sparse cross channel correlation term and sparse gradient term. Due to the 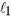-norm penalty of the last two terms, solving this minimization problem does not reduce to a quadratic problem as for the **x**-step. We solve it using a splitting approach that is discussed in detail in the Supplementary Methods, along with comparisons to state-of-the-art methods demonstrating that our method converges to a significantly better optimum in drastically less computational time.

### Fabrication

Encoded lenses are fabricated on Fused Silica substrates by the combination of photolithography and Reactive Ion Etching (RIE) techniques. In the photolithography step, pre-designed patterns are transferred from a photomask to a photoresist layer on the substrate using UV light exposure. In the following RIE step, a certain amount of material in the exposed areas on the substrate is removed by chemically reactive plasma. By iteratively applying this process, multi-level microstructures are formed on the substrates.

Four inch Fused Silica wafers with 0.5 *mm* thickness are used as the substrates. In each fabrication cycle, a 200 *nm* Cr layer is first deposited on the wafer. A 0.6 *μm* photoresist layer (AZ1505) is then spin-coated on the Cr layer and gains its shape after a softbake process (120 °C for 60 *s*). The designed patterns are transferred from the photomask to the photoresist under ultraviolet (UV) light exposure. After exposure, the chemical property of the exposed area on the photoresist changes and can consequently be removed in the developer (MIL AZ726) for 20 seconds. Subsequently, the open area of the Cr layer is removed in Cr etchant and the patterns are transferred to the wafer. In the RIE step, the material (SiO_2_) in the open area is removed by a mixture of Argon and SF_6_ plasma. Each fabrication cycle doubles the number of microstructure levels on the previous profile. Repeating this cycle by 4 iterations, 16 levels of microstructures are created on the wafer. The total etching depth is 1195 *nm* for 16 levels. A final Cr layer is deposited and etched to cover the outer area of the lens for prevention of stray light.

## Additional Information

**How to cite this article**: Heide, F. *et al.* Encoded diffractive optics for full-spectrum computational imaging. *Sci. Rep.* **6**, 33543; doi: 10.1038/srep33543 (2016).

## Supplementary Material

Supplementary Information
