# Supplementary material for: Encoded diffractive optics for full-spectrum computational imaging
Source: Sci Rep. 2016 Sep 16;6:33543. doi: 10.1038/srep33543 (PMC5025844; doi:10.1038/srep33543)
Supplement: Supplementary Information [file srep33543-s1.pdf]

Encoded Diffractive Optics for Full-spectrum Computational Imaging

Supplementary Information

Felix Heide[2,1], Qiang Fu[1], Yifan Peng[2,1] & Wolfgang Heidrich[1]

[1]King Abdullah University of Science and Technology,
[2]The University of British Columbia

Supplementary Methods

We show in this Supplementary Information the details of the design and implementation methods of our encoded lenses. We first introduce the imaging framework of such diffractive imaging systems. The detailed complex matrix factorization algorithm is presented. In the deconvolution step, we illustrate the methods for jointly estimation of the spatially varying PSFs and the latent sharp image. Additional imaging results for different applications using the proposed encoded lenses are also shown. We analyze and evaluate the performance of this system from the perspectives of computational efficiency and image quality at last.

1 Imaging Framework

In general, different spectral distributions of the incident measured illumination on the sensor cause spatially varying Point Spread Functions (PSFs) for a diffractive lens. So, due to varying illuminations or material properties, the PSF $\mathbf{x}_c, (c \in \{1,2,3\})$ for colour channel c is actually an integral of spectral PSFs weighted by the spectral response $\xi_c(\lambda)$ over a spectral range $[\lambda_1, \lambda_2]$

$$\mathbf{x}_c = \int_{\lambda_1}^{\lambda_2} \xi_c(\lambda) x(\lambda) d\lambda, \tag{1}$$

where the spectral PSFs $x(\lambda)$ could be obtained by Fresnel diffraction propagation [Goodman 2005]. We can assume that material and illumination properties are spatially low-frequency. Thus, in a local neighborhood, we can assume that the image captured on the sensor $\mathbf{j}$ is the latent image $\mathbf{v}$ convolved with a spatially invariant PSF $\mathbf{x}$ in each colour channel c, i.e.

$$\mathbf{j}_c = \mathbf{x}_c * \mathbf{v}_c, \tag{2}$$

where $*$ denotes the convolution. In other words, a scene with changing spectral distribution of the colour PSFs would also change accordingly, i.e. the images of points with different spectra in the scene are perceived significantly different on the image plane of a diffractive lens.

Fortunately, when taking an RGB image of the scene with a diffractive lens, we can focus well at least in one channel. This sharp channel is the reference channel in our deconvolution step. The cross-channel correlation between the three channels provides a strong statistical prior that can be used to jointly estimate the spatially-varying, spectrally-dependent PSFs and the underlying latent image.

2 Encoded Lenses

A rotational multi-layer DOE $\mathbf{T}$ can be described as the multiplication of two transmission functions $\mathbf{T}_1$ and $\mathbf{T}_2$ as

$$\begin{aligned}\mathbf{T}(r, \omega - \theta) &= \mathbf{T}_1(r,\omega) \cdot \mathbf{T}_2(r, \omega-\theta) \\ &= \exp\left(i\mathbf{\Phi}_1(r,\omega)\right) \cdot \exp(i\mathbf{\Phi}_2(r,\omega-\theta)) \\ &= \exp\left(i(\mathbf{\Phi}_1(r,\omega) + \mathbf{\Phi}_2(r,\omega-\theta))\right),\end{aligned} \tag{3}$$

where we use the polar coordinates. The phase functions $\mathbf{\Phi}_1$ and $\mathbf{\Phi}_2$ are the respective phase profiles of the two DOEs. The second term has been rotated by θ to encode different focal lengths. We optimize the two DOEs for a continuous angle range at 6 discrete angles for our encoded lenses. The focal lengths are inversely proportional to the rotation angles, i.e. for 0 rotation angle, the focal length is at infinity, and the optical power (the reciprocal of focal length) is a linear function of the rotation angle. For the special case of a Fresnel lens, given a target function $\mathbf{T}$, one can find an approximation as done by[Bernet and Ritsch-Marte 2008; Bernet et al. 2013]. However, the design of arbitrary target transmission functions $\mathbf{T}$ is challenging and the accuracy of their approximation is limited. We choose to reformulate the problem from above as a matrix factorization problem. This is possible by remapping polar coordinates (r,ω) to linear indices addressing the columns of two complex matrices $\mathbf{A}$ and $\mathbf{B}$.

$$\begin{aligned}\mathbf{A}_{\text{opt}}, \mathbf{B}_{\text{opt}} &= \underset{\mathbf{A}\in\mathbb{C}^{m\times r}_{|\cdot|=1}, \mathbf{B}\in\mathbb{C}^{n\times r}_{|\cdot|=1}}{\text{argmin}} \ \frac{1}{2}\left\|\mathbf{T} - \mathbf{A}\mathbf{B}^\dagger\right\|^2_{\mathbf{W}} \\ &= \underset{\mathbf{A}\in\mathbb{C}^{m\times r}_{|\cdot|=1}, \mathbf{B}\in\mathbb{C}^{n\times r}_{|\cdot|=1}}{\text{argmin}} \ \frac{1}{2}\left\|\mathbf{W}\circ\mathbf{T} - \mathbf{W}\circ\mathbf{A}\mathbf{B}^\dagger\right\|^2_F,\end{aligned} \tag{4}$$

where $\mathbf{B}^\dagger$ is the complex conjugate of the matrix $\mathbf{B}$. In the matrix factorization problem from above, we want to solve for two complex layer matrices $\mathbf{A}_{\text{opt}}, \mathbf{B}_{\text{opt}}$, whose product $\mathbf{A}_{\text{opt}}\mathbf{B}^\dagger_{\text{opt}}$ results in the complex target matrix $\mathbf{T}$. Since the product $\mathbf{A}\mathbf{B}^\dagger$ can potentially encode any combination of front and rear layer pixels, we have added a weighting matrix $\mathbf{W}$ that only selects the ones physically possible over each rotation angle that is desired. Please see [Ho 2008] for a detailed introduction to weighted (non-negative) matrix factorization methods. For static elements (that are not changed over time), the matrices $\mathbf{A}, \mathbf{B}$ are of rank 1. If they are changed over time during the exposure (e.g. with a digital phase modulator), higher rank factorizations could be realized. Since the optimization is itself a non-convex problem, the two layer distributions we find is not a unique solution, but one of the feasible solutions.

Note that modeling Eq. (3) as a factorization problem is very general and can also solve any physical setup (not only rotations, but also shift etc as well as time-multiplexing). Furthermore, more than two DOEs can also be supported as a straightforward Tensor extension. By using complex numbers we also allow to include attenuation terms in our design. However, if we don't want our elements to contain any attenuation, we end up not with an unconstrained, but a constrained optimization problem where the absolute value of each complex matrix element is one (denoted as $\mathbb{C}_{|\cdot|=1}$). We will see below that this is not a computational issue.

2.1 Complex Constrained Rank-1 Factorization

Note that in our case the rank of the two matrices is 1 (since we only have two static DOEs) such that Eq. (4) can be simplified to

$$\mathbf{a}_{\text{opt}}, \mathbf{b}_{\text{opt}} = \underset{\mathbf{a}\in\mathbb{C}^{m}_{|\cdot|=1}, \mathbf{b}\in\mathbb{C}^{n}_{|\cdot|=1}}{\text{argmin}} \ \frac{1}{2}\left\|\mathbf{T} - \mathbf{a}\mathbf{b}^\dagger\right\|^2_{\mathbf{W}}. \tag{5}$$

We now solve the bi-convex matrix factorization problem using the following alternating least squares method in Algorithm 1.

Note that the constraints are gone in Alg. 1, since each of the two constrained biconvex optimization problems optimize for isotropic norms. Thus, the constraint can simply be expressed as a renormalization after solving the **b-step** and **a-step** subproblems. Both subproblems are solved using fast Newton updates, which can be done very efficiently since the resulting Hessian matrix turns out to be diagonal.

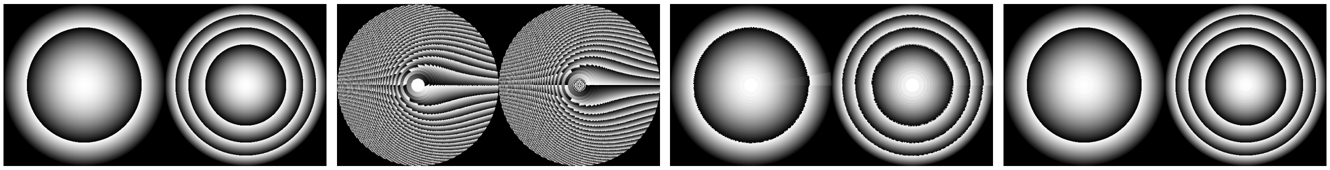

(a) Target phase function for two different angles

(b) Our factorized phase plates for the target in **(a)**

(c) Simulated target using analytical phase plates from [Bernet et al. 2013]

(d) Simulated target using our factorized phase plates from **(b)**

Supplementary Figure 1: Example of our factorization-based lens design. *The target phase functions for two different angles (a) and the factorization result of the lens (b) found by our complex factorization method are shown. (c) shows a simulation of the target using the analytical patterns from [Bernet et al. 2013]. Note especially the step-edge behavior in the phase profile, which is introduced by their analytical rounding design. These artefacts from the analytical design can be removed by our numerically optimized design (d).*

Algorithm 1 Unconstrainted Rank-1 Alternating Least Squares Weighted Complex Matrix Factorization

$k = 0, \mathbf{a}^0_{\text{opt}} = \mathbf{a}_{init}, \mathbf{b}^0_{\text{opt}} = \mathbf{b}_{init}$
repeat
  $\mathbf{b}^{k+1}_{\text{opt}} := \operatorname{argmin}_{\mathbf{b}} \frac{1}{2} \left\| \mathbf{T} - \mathbf{a}\mathbf{b}^{\dagger} \right\|^2_{\mathbf{W}}$ ▷ **b-step**
  $\mathbf{b}^{k+1}_{\text{opt}} := \frac{\mathbf{b}^{k+1}_{\text{opt}}}{|\mathbf{b}^{k+1}_{\text{opt}}|}$
  $\mathbf{a}^{k+1}_{\text{opt}} := \operatorname{argmin}_{\mathbf{a}} \frac{1}{2} \left\| \mathbf{T} - \mathbf{a}\mathbf{b}^{\dagger} \right\|^2_{\mathbf{W}}$ ▷ **a-step**
  $\mathbf{a}^{k+1}_{\text{opt}} := \frac{\mathbf{a}^{k+1}_{\text{opt}}}{|\mathbf{a}^{k+1}_{\text{opt}}|}$
  $k := k + 1$
until Optimality achieved

2.1.1 Newton Updates

Let us consider the **b-step**; the derivations for **a-step** follow from symmetry. We now have

$$\begin{aligned} \mathbf{b}_{\text{opt}} &= \operatorname*{argmin}_{\mathbf{b}} \frac{1}{2} \left\| \mathbf{T} - \mathbf{a}\mathbf{b}^{\dagger} \right\|^2_{\mathbf{W}} \\ &= \operatorname*{argmin}_{\mathbf{b}} \underbrace{\frac{1}{2} \left\| \operatorname{diag}(\mathbf{W})\,\mathbf{t} - \operatorname{diag}(\mathbf{W})\,\mathbf{O_a}\mathbf{b} \right\|^2_2}_{f(\mathbf{b})}, \end{aligned} \tag{6}$$

where $\operatorname{diag}((\cdot))$ puts the matrix from the subscript on the diagonal and $\mathbf{O}_{(\cdot)}$ corresponds to the outer vector product operation with the vector in the subscript and the right hand side, followed by vectorization. Having reformulated f in Eq. (6), we can easily derive the gradient

$$\nabla f = \mathbf{O}^{\dagger}_{\mathbf{a}} \operatorname{diag}(\mathbf{W})\,\mathbf{O_a}\mathbf{b} - \mathbf{O}^{\dagger}_{\mathbf{a}} \operatorname{diag}(\mathbf{W})^2\,\mathbf{t}. \tag{7}$$

The operator $\mathbf{O}^{T}_{\mathbf{a}}$ is the same as the outer vector product operation plus a subsequent summation over the rows of the resulting matrix. For the Hessian we finally get a diagonal matrix with

$$\frac{\partial^2 f}{\partial b^2} = \mathbf{O}^{\dagger}_{\mathbf{a}} \operatorname{diag}(\mathbf{W})\,\mathbf{O_a}. \tag{8}$$

In Newton's method we can exploit the structure of our problem. Since the Hessian is a diagonal, the matrix inversion in Newton's method becomes a point-wise division:

Algorithm 2 Newton Update to solve the **b-step**

repeat
  $\mathbf{b}^{k+1}_{\text{opt}} := \mathbf{b}^{k+1}_{\text{opt}} - \frac{\nabla f}{\frac{\partial^2 f}{\partial b^2}}$ ▷ Pointwise division
  $k := k + 1$
until Optimality achieved

2.2 Comparisons

Having formulated the optimization method for the design of our encoded lenses, we show a few comparison examples of regular Fresnel lens designs using different methods in Supplementary Table 1. We have also included an example of the analytical solution [Bernet and Ritsch-Marte 2008] for comparison. Our approach outperforms the analytical and all other state-of-the-art methods significantly, both in terms of objective value and running time. The results for the proposed parameter settings of all methods are shown. The lower objective value leads to significantly improved phase transition reconstructions. Supplementary Fig. 1 shows a Fresnel lens example, specifically for which [Bernet and Ritsch-Marte 2008] proposed analytical phase patterns. Even in this case our method provides better transition curves without discrete levels. Note that our method in principle can encode any lens design and geometric arrangement that is expressible by a phase function.

Supplementary Table 1: Convergence of the rank-1 complex matrix factorization. *We consider a weighted rank-1 factorization problem in Supplementary Fig. 1. Both, state-of-the-art weighted factorization algorithms [Xu and Yin 2013; Haeffele et al. 2014] as well as the analytical design from [Bernet and Ritsch-Marte 2008] are compared here. We have run all optimization methods till convergence and compare here the final objective value and running time.*

Method	Objective	Time till convergence (s)
Analytical [Bernet and Ritsch-Marte 2008]	1.35×10^5	-
Block-Coordinate LRA [Xu and Yin 2013]	6.81×10^4	1.0×10^3
Structured LRA [Haeffele et al. 2014]	6.82×10^4	1.0×10^3
Alternating Projections [Markovsky 2011]	7.10×10^4	5.0×10^3
Ours	$\mathbf{4.94 \times 10^1}$	$\mathbf{1.2}$

2.3 Diffraction efficiency

In the design of the encoded lens, we optimize the complex transmission functions of the target lenses directly by complex matrix factorization. Therefore, we don't have the problems that arise in [Bernet and Ritsch-Marte 2008; Bernet et al. 2013], in which the two-sector phase profiles and round errors are sources that degrade the diffraction efficiency. However, the diffraction efficiency of our encoded lens also changes with rotation angles, as illustrated in Supplementary Figure 2 for the design with focal length $(-\infty, -50mm] \cup [50mm, \infty)$. The diffraction efficiency is calculated through Fresnel diffraction propagation. For each rotation

angle, we calculate the axial intensities on the focal plane for our optimized blazed encoded lens and the fabricated 16-level encoded lens. The intensity for the corresponding ideal lenses with the same focal lengths are calculated as the reference intensity. Here we show not only the design angles, but also angles in between. The diffraction efficiency may fall off for some in-between angles. However, this fall-off can be improved by increasing the number of optimization angles easily by our algorithm. As shown here, optimizing at 6 angles already shows good diffraction efficiency for even more angles (12 angles) sampled in the design range.

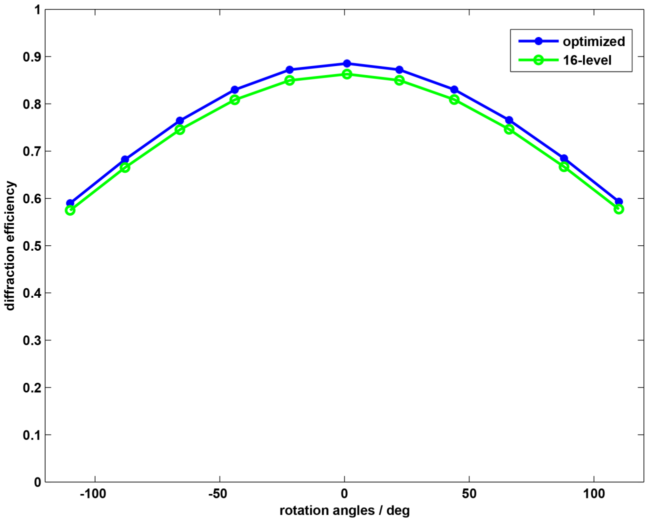

Supplementary Figure 2: Diffraction efficiency subject to rotation angles. *For the design with focal length* $(-\infty, -50mm] \cup [50mm, \infty)$*, we show the diffraction efficiency for the design angles as well as the angles in between. The diffraction efficiency peaks at the zero rotation angle and falls off gradually for larger rotation angles.*

3 Reconstruction

This section covers the computational framework which enables us to remove the severe wavelength-dependent aberrations from our DOEs. We present a novel image reconstruction method that jointly estimates the underlying scene-dependent, spatially-varying PSFs and the latent sharp image. Since the PSFs are estimated jointly with the image, our method can be understood as a self-calibrating reconstruction method that adapts to the scene. We first describe the overall reconstruction framework and the sub-problem specific to the PSF estimation. Subsequently, the second part covers an efficient way to solve the non-blind deconvolution sub-problem of the joint reconstruction method.

Our joint method outperforms all state-of-the-art blind estimation methods in simulation and real-world measurements. It furthermore is computationally efficient, which is key in handling the extremely large PSFs caused by DOEs. Since handling noise and large PSFs is a central issue in blind and non-blind deconvolution, we have formulated a method that addresses both of these issues and at the same time maintains high computational efficiency.

3.1 Blind Deconvolution

The PSFs for our DOEs change based on the reflectance of the objects and illumination spectra in the scene. Having ensured that our optics focus at least well in one channel, we can solve for the PSFs exploiting cross-channel correlation between the channels. In particular we solve the following optimization problem:

$$\mathbf{x}_{\text{opt}} = \underset{\mathbf{x},\mathbf{v}}{\text{argmin}} \; \|\mathbf{V}\mathbf{x} - \mathbf{j}\|_2^2 + \alpha \sum_{a=1}^{3} \|\mathbf{H}_a \mathbf{x}\|_2^2 + \beta \|\mathbf{x}\|_2^2 + \gamma \sum_{a \neq r} \|\mathbf{H}_a \mathbf{v} - \mathbf{H}_a \mathbf{i}_r\|_1 + \mu \sum_{a} \|\mathbf{H}_a \mathbf{v}\|_1, \tag{9}$$

The matrix $\mathbf{V}$ expresses here the convolution with the latent image $\mathbf{v}$. That means the results of the matrix-vector product $\mathbf{V}\mathbf{x}$ is the same as the vectorized convolution $\mathbf{v} * \mathbf{x}$. Note that Eq. (9) is the matrix-notation equivalent of Eq. 5 of the main draft. Convolution is a symmetric operator, hence it can be interchangeably expressed as either $\mathbf{V}\mathbf{x}$ or $\mathbf{X}\mathbf{v}$, where $\mathbf{V}$ and $\mathbf{X}$ are the corresponding convolution matrices. The operators $\mathbf{H}_a$ $(a = 1, 2, 3)$ are first order spatial gradient filters for RGB channels, and $\mathbf{i}_r$ is a sharp image in reference channel r.

In the first row we have a classical ℓ_2 dataterm and gradient priors on the PSF $\mathbf{x}$. However, this simple term is not sufficient to get decent enough blind estimation quality. That is why recent approaches use complex priors on the latent image, such as cluster colour distributions [Lai et al. 2015], patch based priors [Sun et al. 2013], [Michaeli and Irani 2014] and shock filters [Schuler et al. 2012]. This means they are actually not just solving a simple quadratic problem like the one in the first line of Eq. (9), but a complex sequence of individual heuristic steps. See [Perrone and Favaro 2014] for a discussion of different heuristics behind blind deconvolution.

Cross-channel Prior In our approach, we add a cross-channel term on the latent image $\mathbf{v}$ in the second row of Eq. (9). This cross-channel term is derived from prior statistical knowledge about the correlation between different spectral bands. Similar to [Heide et al. 2013], we model gradient differences with a heavy-tailed prior:

$$p(\mathbf{x}_{\text{opt}} | \gamma, \mathbf{i}_r) \propto \exp\left(-\gamma \sum_{a} \|\mathbf{H}_a \mathbf{v} - \mathbf{H}_a \mathbf{i}_r\|_1\right). \tag{10}$$

The key observation of our cross-channel prior is that for a latent (sharp) image, changes in chroma and luma are sparsely distributed in natural images. That means, in most areas the gradients of different channels are close, except for few large changes in chroma and luma (e.g. commonly at object or material boundaries). Note, that this model differs from the one proposed in [Heide et al. 2013]. The authors assume only chroma changes to be sparse. The downside is that this leads to severe instabilities of the prior term for low intensities. Furthermore, we will see below that our formulation leads to a drastically improved convergence and overall run time.

To verify our model, we have done a statistical evaluation of 10,000 images from the Imagenet data set [Deng et al. 2009]. Figure 4 shows the empirical distribution of the cross-channel gradients $\mathbf{v}_c = \mathbf{H}_a \mathbf{v} - \mathbf{H}_a \mathbf{i}_r$ accumulated for all images, where $\mathbf{v}$ are here the red and blue channel and $\mathbf{i}_r$ is the green channel. The plot also shows a Gaussian fit $p(\mathbf{v}_c) \propto e^{-\gamma|\mathbf{v}_c|^2}$, Laplacian fit $p(\mathbf{v}_c) \propto e^{-\gamma|\mathbf{v}_c|}$ and Hyper-Laplacian $p(\mathbf{v}_c) \propto e^{-\gamma|\mathbf{v}_c|^{\frac{2}{3}}}$. We can see that the empirical chross-channel gradient distibution is heavy-tailed and thus not well approximated by a normal distribution. While a Hyper-Laplacian fit best models the underlying distribution, the Laplacian is the best *convex* relaxation of the fitted

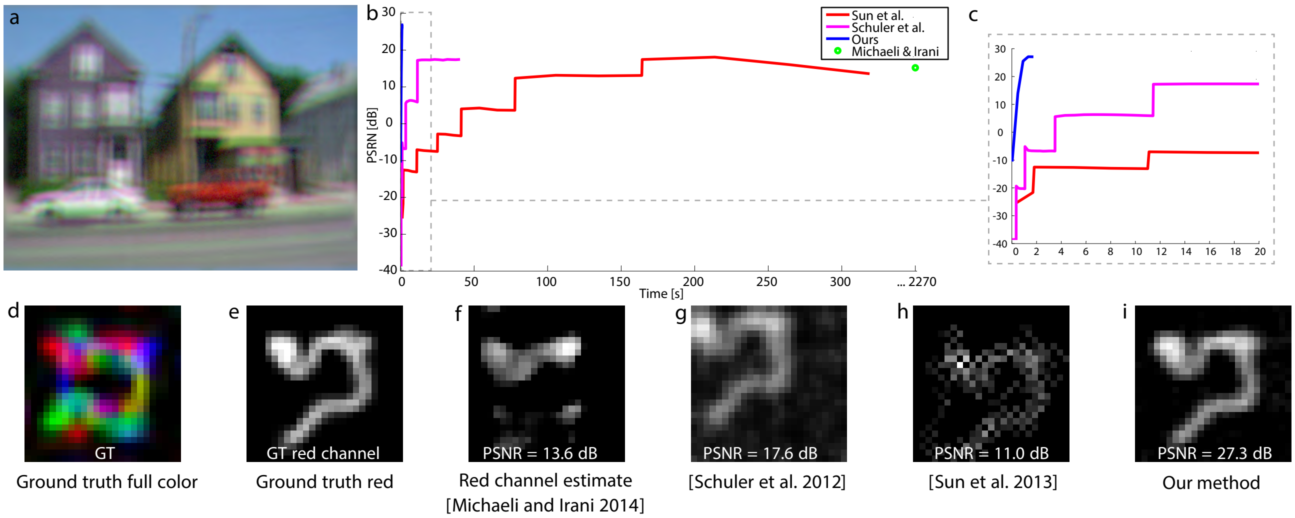

Supplementary Figure 3: Convergence of our blind deconvolution method. *(a) The blurred and noisy input image considered in this numerical example. The original image is part of the LabelMe dataset [Russell et al. 2008]. (b) shows the empirical convergence of our method compared to state-of-the-art methods. We visualize the PSNR as distance to the ground truth PSF here. Our method is plotted at the very left of this plot. The plot in (c) shows a zoom-in of the first few seconds from the plot in (b). From (d) to (i) compares blind PSF estimates of our method versus the state-of-the-art.*

distribution. We therefore choose in Eq. (10) the Laplacian distribution as the best convex fit.

Finally, the cross-channel term in Eq. (9) follows from Maximum-a-Posteriori (MAP) Estimation with the prior from Eq. (10).

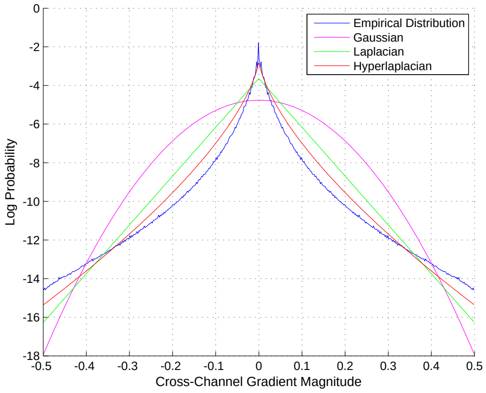

Supplementary Figure 4: Empirical Cross-Channel Statistics on a large data set. *Cross-channel gradients have been accumulated from 10,000 randomly selected images from the Imagenet data set [Deng et al. 2009]. The empirical distribution follows a heavy-tailed distribution that can be well approximated with a Laplacian as best convex relaxation of the non-convex Hyper-Laplacian fit.*

Algorithm The exciting thing about adding the cross-channel prior is that enables us to actually simply solve the bi-convex problem from Eq. (9) using coordinate descent **without any additional priors or optimization schedule tricks**. The full alternating coordinate descent method is given in Algorithm 3.

In Algorithm 3 both sub-problems contain simple linear operators $\mathbf{X}^k$ and $\mathbf{V}^k$ that represent convolutions. This formulation can be used since convolutions are commutative:

$$\mathbf{V}\mathbf{x} = \mathbf{X}\mathbf{v} \quad \text{since} \quad \mathbf{x} * \mathbf{v} = \mathbf{v} * \mathbf{x} \tag{11}$$

Algorithm 3 Blind PSF Estimation Using Coordinate Descent

$\mathbf{x}^0 = \delta, \mathbf{v}^0 = \mathbf{j}$

repeat

$$\mathbf{x}^{k+1} = \underset{\mathbf{x}}{\text{argmin}} \ \sum_a \|\mathbf{V}^k\mathbf{x} - \mathbf{j}\|_2^2 + \alpha \sum_a \|\mathbf{H}_a\mathbf{x}\|_2^2 + \beta\|\mathbf{x}\|_2^2 \qquad \triangleright \ \textbf{x-step}$$

$$\mathbf{v}_i^{k+1} = \underset{\mathbf{v}}{\text{argmin}} \ \sum_a \|\mathbf{X}^k\mathbf{v} - \mathbf{j}\|_2^2 + \gamma \sum_a \|\mathbf{H}_a\mathbf{v} - \mathbf{H}_a\mathbf{i}_r\|_1 + \mu\|\mathbf{H}_a\mathbf{v}\|_1 \qquad \triangleright \ \textbf{v-step}$$

$k := k + 1$

until Optimality achieved

The optimization from Algorithm 3 solves the bi-convex minimization problem from Eq. (9) via coordinate descent. That means we keep one of the two variables $\mathbf{x}, \mathbf{v}$ fixed at a time while minimizing the objective with respect to the other variable in an alternating fashion. This approach leads to the two sub-problems from the steps **x-step** and **v-step** of algorithm Algorithm 3, both of which are now much easier to solve than the joint objective from Eq. (9). This is already easy to see, since all of these sub-steps are now convex optimization problems, while the joint objective is non-convex.

$$\begin{aligned} \mathbf{V}' = \mathbf{V}\mathbf{E} \quad &\text{and} \quad \mathbf{x}' = \mathbf{E}^{-1}\mathbf{x} \quad \text{since} \\ \mathbf{V}'\mathbf{x}' &= \mathbf{V}\mathbf{E}\mathbf{E}^{-1}\mathbf{x} = \mathbf{V}\mathbb{I}\mathbf{x} \end{aligned} \tag{12}$$

Having transformed out non-convex problem into a sequence of convex sub-problems, we can derive efficient solutions for these individual sub-problems.

Solving the x-step The **x-step** can be solved efficiently as follows

$$
\mathbf{x}_{\text{opt}} = \underset{\mathbf{x}}{\text{argmin}} \; \underbrace{\|\mathbf{V}\mathbf{x} - \mathbf{j}\|_2^2 + \alpha \sum_a \|\mathbf{H}_a \mathbf{x}\|_2^2 + \beta \|\mathbf{x}\|_2^2}_{\chi(\mathbf{x})}
$$

$$
\begin{aligned}
&\Leftrightarrow \frac{\partial \chi(\mathbf{x}_{\text{opt}})}{\partial \mathbf{x}} = 2\left(\mathbf{V}^T\mathbf{V}\mathbf{x}_{\text{opt}} - \mathbf{V}^T\mathbf{j}\right) + \\
&\qquad \alpha \sum_a 2\mathbf{H}_a^T\mathbf{H}_a\mathbf{x}_{\text{opt}} + 2\beta\mathbf{x}_{\text{opt}} \overset{!}{=} 0 \\
&\Leftrightarrow \mathbf{V}^T\mathbf{V}\mathbf{x}_{\text{opt}} + \alpha \sum_a \mathbf{H}_a^T\mathbf{H}_a\mathbf{x}_{\text{opt}} + \beta\mathbf{x}_{\text{opt}} = \mathbf{V}^T\mathbf{j} \\
&\Leftrightarrow \left(\mathbf{V}^T\mathbf{V} + \alpha \sum_a \mathbf{H}_a^T\mathbf{H}_a + \beta\mathbb{I}\right)\mathbf{x}_{\text{opt}} = \mathbf{V}^T\mathbf{j} \\
&\Leftrightarrow \mathbf{x}_{\text{opt}} = \mathcal{F}^{-1}\left(\frac{\mathcal{F}(\mathbf{V})^*\mathcal{F}(\mathbf{j})}{\mathcal{F}(\mathbf{V})^*\mathcal{F}(\mathbf{V}) + \alpha\sum_a \mathcal{F}(\mathbf{H}_a)^*\mathcal{F}(\mathbf{H}_a) + \beta}\right)
\end{aligned} \tag{13}
$$

Minimizing the quadratic from the $\mathbf{x}$-**step** is equivalent to solving the linear equation system from the second last row in Eq. (13). The linear system is composed of the matrices $\mathbf{V}$, $\mathbf{H}$ and $\beta\mathbb{I}$ which all are very structured, that is all of these matrices are convolution matrices. This structure can be exploited by reformulating this linear equation system in the frequency domain in the last row of Eq. (13). The system can then be inverted very efficiently by pointwise division.

Solving the $\mathbf{v}$-step The $\mathbf{v}$-**step** of our blind deconvolution Algorithm 3 requires the solution of a deconvolution problem with known kernel $\mathbf{X}^k$. It involves a quadratic data term, sparse cross channel correlation term and sparse gradient term. Due to the ℓ_1-norm penalty of the last two terms solving this minimization problem does not reduce to a quadratic problem as for the $\mathbf{x}$-**step** in Eq. (13). We solve it with a splitting method that is discussed in detail in the following subsection on Non-Blind Deconvolution.

Note that (given a kernel estimate) solving the *non-blind deconvolution problem is in fact part of our joint blind reconstruction*. This is in strong contrast to all recent state-of-the-art blind deconvolution methods such as [Michaeli and Irani 2014; Sun et al. 2013].

Convergence and Comparisons We have compared the blind PSF estimation method from Algorithm 3 to the most recent state-of-the-art blind deconvolution methods in both simulation and using real measurements. While we evaluate our full reconstruction method using real measurements later in this document, we illustrate the impact of our method with the example in Supplementary Fig. 3. This figure demonstrates the benefit of using cross-channel information for large kernels and severe noise levels, where recent blind estimation approaches fail. The algorithm parameters of the methods we compare to have been tuned for best performance.

All of our empirical convergence experiments show that our approach **converges to a significantly better optimum in drastically less computational time (and iterations)**. The convergence plots in the top right of the figure demonstrate that we outperform recent state-of-the-art methods [Michaeli and Irani 2014; Sun et al. 2013; Schuler et al. 2012] significantly. Note that the plot in the center shows our method on the very left close to the left of the plot. We therefore added a zoom-in right next to it. The optimum that our method finds is significantly better than all compared methods as shown in the bottom row. In terms of Peak Signal to Noise Ratio (PSNR) it is **almost 9.7 dB better than the best of all compared methods**.

3.2 Non-Blind Deconvolution

The previous subsection has described our overall reconstruction Algorithm 3 that jointly self-calibrates the PSF and estimates the unknown latent image. This subsection explains how we solve the non-blind deconvolution sub-problem ($\mathbf{v}$-**step**), that is solving for the latent image given a fixed PSF estimate $\mathbf{X}^k$ and prior knowledge of spectral gradient correlation. The specific choice of the cross-channel prior from Sec. 3.1 will allow us to formulate a very efficient inversion method. To show this, it makes sense to first look at the formulation proposed by [Heide et al. 2013]. The authors solve the following optimization problem for one channel c of the three considered channels:

$$
\begin{aligned}
\mathbf{v}_{\text{opt}} = \underset{\mathbf{v}}{\text{argmin}} \; & \|\mathbf{X}\mathbf{v} - \mathbf{j}\|_2^2 + \mu \sum_a \|\mathbf{H}_a\mathbf{v}\|_1 + \\
& \gamma \sum_a \|\mathbf{H}_a\mathbf{v} \cdot \mathbf{i}_r - \mathbf{H}_a\mathbf{i}_r \cdot \mathbf{v}\|_1,
\end{aligned} \tag{14}
$$

The authors introduced slack variables for all the terms $\mathbf{H}_a\mathbf{x}$ and all the $\mathbf{H}_a\mathbf{v} \cdot \mathbf{i}_r - \mathbf{H}_a\mathbf{i}_r \cdot \mathbf{v}$ terms. Two slack variables are necessary since statistics on normalized gradients are considered (multiplication with $\mathbf{i}_r$ and $\mathbf{v}$). The resulting optimization problem is then solved via Chambolle and Pock's primal-dual framework [Chambolle and Pock 2011]. Introducing many slack terms results in slow convergence in order to achieve consensus between all the consensus constraints and the objective terms. Therefore [Heide et al. 2013] method needs around 300 iterations for convergence, which makes it prohibitive for most real-world applications.

3.2.1 Efficient Optimization

Our formulation of the cross-channel prior leads to the following sub-problem ($\mathbf{v}$-**step** of Algorithm 3):

$$
\begin{aligned}
\mathbf{v}_{\text{opt}} = \underset{\mathbf{v}}{\text{argmin}} \; & \|\mathbf{X}\mathbf{v} - \mathbf{j}\|_2^2 + \mu \sum_a \|\mathbf{H}_a\mathbf{v}\|_1 + \\
& \gamma \sum_a \|\mathbf{H}_a\mathbf{v} - \mathbf{H}_a\mathbf{i}_r\|_1,
\end{aligned} \tag{15}
$$

We will show that this formulation will only require a single slack variables for $\mathbf{H}_a\mathbf{v}$. That is the same number of slack variables usually needed for ℓ_1-deconvolution methods. To see this, let us formulate the proximal operators that would be necessary if we were to apply [Chambolle and Pock 2011] to Eq. (15). Two proximal operators, one for the sparse gradient term and the other for the cross-channel term, can be formulated using only $\mathbf{p} = \mathbf{H}_a\mathbf{v}$ as variable:

$$
\begin{aligned}
&\mathbf{prox}_{\theta\|\cdot\|_1}(\mathbf{p}) = \max\left(1 - \frac{\theta\beta}{|\mathbf{p}|}, 0\right) \odot \mathbf{p} \quad \textbf{Shrinkage} \\
&\mathbf{prox}_{\theta\|\cdot-\alpha\|_1}(\mathbf{p}) = \underset{\mathbf{x}}{\text{argmin}} \; \theta\|\mathbf{x} - \alpha\|_1 + \frac{1}{2}\|\mathbf{x} - \mathbf{p}\|_2^2 \\
&= \alpha + \underset{\mathbf{z}}{\text{argmin}} \; \theta\|\mathbf{z}\|_1 + \frac{1}{2}\|\mathbf{z} + \alpha - \mathbf{p}\|_2^2 \quad \text{with} \quad \mathbf{z} := \mathbf{x} - \alpha \\
&= \alpha + \underset{\mathbf{z}}{\text{argmin}} \; \theta\|\mathbf{z}\|_1 + \frac{1}{2}\|\mathbf{z} - (\mathbf{p} - \alpha)\|_2^2 \\
&= \mathbf{prox}_{\theta\|\cdot\|_1}(\mathbf{p} - \alpha) + \alpha \\
&= \max\left(1 - \frac{\theta\beta}{|\mathbf{p} - \alpha|}, 0\right) \odot \mathbf{p} + \alpha \qquad \textbf{Cross-shrinkage}
\end{aligned} \tag{16}
$$

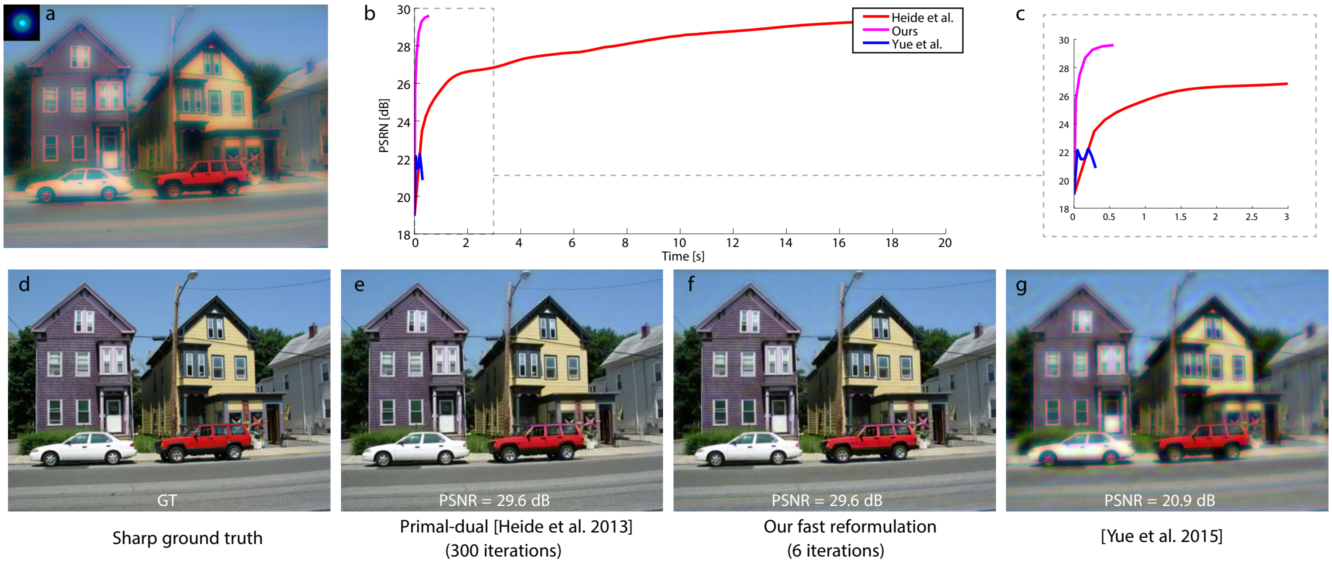

Supplementary Figure 5: Convergence of our non-blind deconvolution method. *(a) shows the blurred and noisy input image and the corresponding, extremely large, chromatic PSF in the top left. The original image (d) is part of the LabelMe dataset [Russell et al. 2008]. (b) shows the empirical convergence of our method compared to the state-of-the-art methods. Our method is plotted at the very left of this plot. A zoom-in for the first few seconds is shown in (c). The results (e) to (g) compare our method against the state-of-the-art.*

where here $\alpha = \mathbf{H}_a \mathbf{i}_r$. To derive the second proximal operator, we have used a simple substitution trick via substitution of $\mathbf{z} := \mathbf{x} - \alpha$. The proximal operator then reduces to the shrinkage operator. Using this trick, we have defined the proximal operators for both, gradient and cross channel term, using the same slack variable for $\mathbf{p} = \mathbf{H}_a \mathbf{x}$. It is easy to see, that for Eq. (14) the multiplicative term in the cross-channel term prohibits this approach and thus another slack variable needs to be introduced.

Note that we give here the proximal operators for the primal functions. The proximal operators for their convex conjugates can be easily computed by using Moreau's identity [Boyd et al. 2011].

Having defined the objective in a way that it possesses an efficient splitting, we can now choose between different proximal algorithms to minimize it. Below, we first formulate a generalized form of the objective that fits into most recent proximal algorithms. Subsequently, we give detailed derivations for two choices, the well known Alternating Direction Method of Multiplier (ADMM) and the a very fast approximation using fast quadratic splitting.

3.2.2 Generalized Objective

The objective of (15) can be generalized to a sum of penalties f_i on linear transforms $\mathbf{K}(i)\mathbf{z}$ with $\mathbf{z}$ being the unknown now:

$$\underset{\mathbf{z}}{\operatorname{argmin}} \sum_{i=1}^{I} f_i\left(\mathbf{K}(i)\mathbf{z}\right) \quad \text{with} \quad \mathbf{K} = \begin{bmatrix} \mathbf{K}_1 \\ \vdots \\ \mathbf{K}_h \end{bmatrix}, \qquad (17)$$

where here $\mathbf{K} \in \mathbb{R}^{a \times n}$ is one large matrix that is composed of stacked linear operators $\mathbf{K}_{1\ldots h}$. The linear operator $\mathbf{K}(i) \in \mathbb{R}^{a_i \times n}$ selects now a subset of a_i rows of $\mathbf{Kz}$. This subset of rows is then the input for the penalty functions $f_i : \mathbb{R}^{a_i} \to \mathbb{R}$, which are closed, proper, convex functions. The formulation from Eq. (17) is similar to [Heide et al. 2015], which considers the specific application of Convolutional Sparse Coding. Note however, the difference in the definition of $\mathbf{K}(i)$, which is here more generalized. We can now frame our problem from Eq. (15) in the more general form by setting:

$$\begin{aligned} \mathbf{v}_{\text{opt}} &= \underset{\mathbf{v}}{\operatorname{argmin}} \ \|\mathbf{Xv} - \mathbf{j}\|_2^2 + \mu \sum_a \|\mathbf{H}_a \mathbf{v}\|_1 + \\ &\qquad \gamma \sum_a \|\mathbf{H}_a \mathbf{v} - \mathbf{H}_a \mathbf{i}_r\|_1 \\ &= \underset{\mathbf{z}}{\operatorname{argmin}} \ f_1(\mathbf{Xz}) + \sum_{a=1}^{2} f_{(i+1)}(\mathbf{H}_a \mathbf{z}) + f_{(i+3)}(\mathbf{H}_a \mathbf{z}) \qquad (18) \\ &= \underset{\mathbf{z}}{\operatorname{argmin}} \ \sum_{i=1}^{5} f_i\left(\mathbf{K}(i)\mathbf{z}\right), \quad \text{with} \quad \mathbf{K} = \begin{bmatrix} \mathbf{X} \\ \mathbf{H}_1 \\ \mathbf{H}_2 \end{bmatrix}, \quad \text{and} \end{aligned}$$

$$f_1(\mathbf{p}) = \|\mathbf{p} - \mathbf{j}\|_2^2, \quad f_{2,3}(\mathbf{p}) = \mu\|\mathbf{p}\|_1, \quad f_{4,5}(\mathbf{p}) = \|\mathbf{p} - \alpha\|_1$$

Having formulated our problem as a sum of functions operating on the stacked matrix $\mathbf{K}$ we can implement Eq. (17) in existing optimization frameworks, such as Chambolle and Pock's similar to [Heide et al. 2013], or ADMM [Almeida and Figueiredo 2013]. Note, that commonly these frameworks split only a sum of two functions. However, we can simply set one of the two functions to be the sum $f(\cdot) = \sum_{i=1}^{I} f_i(\cdot)$ which will be described below in further detail.

3.2.3 Optimization using ADMM

This subsection explains how to solve Eq. (18) using the alternating direction method of multipliers (ADMM). ADMM solves the following general problem:

$$\underset{\mathbf{z}}{\operatorname{argmin}} \ h(\mathbf{v}) + g(\mathbf{z}) \quad \text{subject to} \quad \mathbf{Kv} = \mathbf{z}. \qquad (19)$$

An in-depth description of ADMM is given in [Boyd et al. 2011]. The most obvious choice to bring our problem into this form is setting $h = 0$ and $g = \sum_{i=1}^{I} f_i(\cdot)$ as mentioned above. However, a

more efficient decomposition is to decompose the set of all functions $\{1, \ldots, I\}$ into $h = \sum_{i \in \Omega} f_i(\cdot)$ and $g = \sum_{i \in \Psi} f_i(\cdot)$ with $\Psi := \{1, \ldots, I\} \setminus \Omega$. The set Ω is here selected to contain all quadratic functions which can be efficiently solved. The unscaled form of ADMM yields then Algorithm 4.

Algorithm 4 ADMM to solve Eq. (18)

1: **for** $k = 1$ to N **do**
2: $\quad \mathbf{v}^{k+1} = \underset{\mathbf{v}}{\operatorname{argmin}} \sum_{i \in \Omega} f_i(\mathbf{v}) + \sum_{j \in \Psi} \|\mathbf{K}_j \mathbf{v} - \mathbf{z}_j + \lambda_j^k\|_2^2$
3: $\quad \mathbf{z}_j^{k+1} = \mathbf{prox}_{\frac{f_j}{\rho}} (\mathbf{K}_j \mathbf{v}_j^{k+1} + \lambda_j^k) \quad \forall j \in \Psi$
4: $\quad \lambda_j^{k+1} = \lambda_j^k + (\mathbf{K}_j \mathbf{v}^{k+1} - \mathbf{z}_j^{k+1}) \quad \forall j \in \Psi$
5: **end for**

Having formulated Algorithm 4, it becomes clear that we only need to derive $|S|$ slack variables for the terms $\mathbf{K}_{i \in \Omega} \mathbf{v}$. Note that this algorithm transforms the joint minimization of the sum of all f_i terms into a sequence of separable minimizations w.r.t. f_i. The quadratic subproblem in Line 2 of Algorithm 4 can be efficiently solved in the Fourier domain (due to our specific choice of Ω) as shown in Eq. (20). To simplify notations we use $\omega_j := \mathbf{z}_j - \lambda_j^k$.

$$
\begin{aligned}
\mathbf{v}_{\text{opt}} &= \underset{\mathbf{v}}{\operatorname{argmin}} \ \frac{1}{2} \sum_{i \in \{1\}} f_i(\mathbf{v}) + \frac{\rho}{2} \sum_{j \in \{2,3\}} \|\mathbf{K}_j \mathbf{v} - \omega_j\|_2^2 \\
&= \underset{\mathbf{v}}{\operatorname{argmin}} \ \underbrace{\frac{1}{2}\|\mathbf{X}\mathbf{v} - \mathbf{j}\|_2^2 + \frac{\rho}{2}\|\mathbf{H}_1 \mathbf{v} - \omega_2\|_2^2 + \frac{\rho}{2}\|\mathbf{H}_2 \mathbf{v} - \omega_3\|_2^2}_{\Omega(\mathbf{v})} \\
\Leftrightarrow \ \frac{\partial \Phi(\Omega \mathbf{v})}{\partial \mathbf{v}} &= (\mathbf{X}^T\mathbf{X} + \rho \mathbf{H}_1^T \mathbf{H}_1 + \rho \mathbf{H}_2^T \mathbf{H}_2)\mathbf{v}_{\text{opt}} - \\
&\quad \mathbf{X}^T \mathbf{j} + \rho \mathbf{H}_1^T \omega_2 + \rho \mathbf{H}_1^T \omega_3 \overset{!}{=} 0 \\
\Leftrightarrow \ (\mathbf{X}^T\mathbf{X} + \rho \sum_{a=1}^{2} \mathbf{H}_a^T \mathbf{H}_a)\mathbf{x}_{\text{opt}} &= \mathbf{X}^T \mathbf{j} + \rho \sum_{a=1}^{2} \mathbf{H}_a^T \omega_{a+1} \\
\Leftrightarrow \ \mathbf{x}_{\text{opt}} &= \mathcal{F}^{-1}\left(\frac{\mathcal{F}(\mathbf{X})^* \mathcal{F}(\omega_1) + \rho \sum_{a=1}^{2} \mathcal{F}(\mathbf{H}_a)^* \mathcal{F}(\omega_{a+1})}{\mathcal{F}(\mathbf{X})^* \mathcal{F}(\mathbf{X}) + \rho \sum_{a=1}^{2} \mathcal{F}(\mathbf{H}_a)^* \mathcal{F}(\mathbf{H}_a)} \right)
\end{aligned} \tag{20}
$$

The remaining parts of Algorithm 4 are the proximal operators in Line 3 and the Lagrange multiplier update in Line 4. Previously, in Eq. (16) we have very efficient point-wise updates for the proximal operators of $\mathbf{prox}_{1/\rho f_\Psi}$. The Lagrange multiplier update from Line 4 is a point-wise operation as well, and therefore very efficiently solvable.

Having defined our specific ADMM Algorithm 4, we note that empirically converged results in about 50 iterations. The primal-dual method from [Chambolle and Pock 2011] performs similar in this case (classical ADMM is a special case of this method). Note that the specific decomposition and substitutions that we made to derive ADMM apply straighforwardly for the derivation of our method using the method from [Chambolle and Pock 2011].

3.2.4 Optimization using Half-Quadratic Splitting

This subsection explains an alternative way to solve the problem in Eq. (18) using Half-Quadratic Splitting [Geman and Yang 1995; Krishnan and Fergus 2009]. This approach will lead to a method with good empirical convergence that can be terminated quickly. In particular, Half-Quadratic Splitting solves the following problem:

$$
\begin{aligned}
\mathbf{v}_{\text{opt}} &= \underset{\mathbf{v}}{\operatorname{argmin}} \ \sum_{i \in \Omega} f_i(\mathbf{K}_i \mathbf{v}) + \sum_{j \in \Psi} f_i(\mathbf{z}_j) + \frac{\rho}{2}\|\mathbf{K}_j \mathbf{v} - \mathbf{z}_j\|_2^2 \\
&= \underset{\mathbf{v},\mathbf{z}}{\operatorname{argmin}} \ \|\mathbf{X}\mathbf{v} - \mathbf{j}\|_2^2 + \mu \sum_a \|\mathbf{z}_a\|_1 + \gamma \|\mathbf{z}_a - \mathbf{H}_a \mathbf{i}_r\|_1 + \\
&\qquad \frac{\rho}{2} \sum_a \|\mathbf{H}_a \mathbf{v} - \mathbf{z}_a\|_2^2,
\end{aligned} \tag{21}
$$

We can see, that auxiliary variables $\mathbf{z}_j$ are introduced here for the terms $\mathbf{K}_j \mathbf{v}$, similarly to the ADMM method. The consensus, however is achieved with a simple quadratic term with a weight ρ. This weight on the constraint is increased during the optimization. It is easy to see that for $\rho \rightarrow \infty$, this new objective from Eq.(21) becomes our original objective from Eq.(15). The method performs a coordinate descent w.r.t. $\mathbf{v}, \mathbf{z}$, while continuously increasing the weight of ρ by a factor α. Algorithm 5 shows the resulting alternating minimization.

Algorithm 5 Half-Quadratic Splitting

1: **for** $k = 1$ to N **do**
2: $\quad \mathbf{v}^{k+1} = \underset{\mathbf{v}}{\operatorname{argmin}} \sum_{i \in \Omega} f_i(\mathbf{K}_i \mathbf{v}) + \rho^k \sum_{j \in \Psi} \|\mathbf{K}_j \mathbf{v} - \mathbf{z}_j\|_2^2$
3: $\quad \mathbf{z}_j^{k+1} = \mathbf{prox}_{\frac{f_j}{\rho}} (\mathbf{K}_j \mathbf{v}_j^{k+1}) \quad \forall j \in \Psi$
4: $\quad \rho^{k+1} = \rho^k * \alpha$
5: **end for**

The subproblem in Line 3 of Alg. 4 can be solved in the frequency domain analogously to the first step in the ADMM method from Eq. (20). The proximal operators are also the same as in the ADMM case and given in Eq. (16).

Consequently, on the first glance, this algorithm looks similar to the ADMM derivation from Alg. 4. However, the key difference is here the Lagrange multipliers in the ADMM have been eliminated here. The use of the Lagrange multipliers allows to have a fixed ρ. In this case we need to scale $\rho \rightarrow \infty$. This scaling can cause both the quadratic step in Line 3 and the proximal operator step in Line 4 to be unstable. Thus, for the Half-Quadratic Splitting method to be stable and solve the convex Eq.(15), it is central to minimize the number of splitting variables and find numerically accurate solutions to both steps. Since we have formulated an analytic solution to the proximal operators in Eq. (16), the method is robust for our problem. The original objective of [Heide et al. 2013], however, needs proper minimization of the consensus constraints and a naive implementation of the Half-Quadratic Splitting does not lead to reasonable results as noted by the authors. The method from [Yue et al. 2015] formulates a lookup-table based inversion for their specific proximal operators which suffers from inaccuracy for large values of β.

Convergence and Comparisons We have compared the Half-Quadratic Splitting method from Algorithm 5 to the state-of-the-art cross-channel methods from [Heide et al. 2013] and [Yue et al. 2015]. Empirical convergence experiments are shown in Supplementary Fig. 5.

Using Half-Quadratic Splitting, we observe that we can achieve almost converged results in 5 iterations, which means a *speedup of* $\times 60$ *compared to [Heide et al. 2013] and* $\times 10$ *compared to ADMM.* Note, that the reformulated proximal operators from Eq. (16) are key here. Note that our Half-Quadratic Splitting approach converges to the same PSNR value. Averaging over all images from the 75 test cases from Supplementary Fig. 3 yields

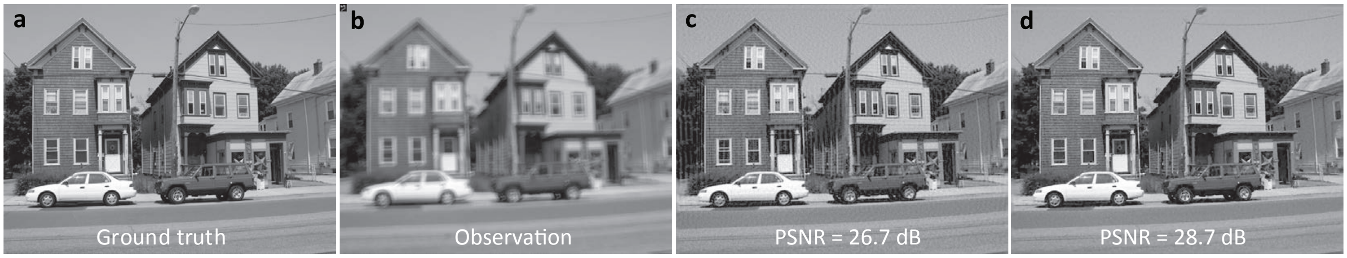

Supplementary Figure 6: Effect of Poisson noise on deconvolution with large kernels. *(a) Sharp ground truth taken from the LabelMe dataset [Russell et al. 2008], (b) Blurred observation corrupted by Poisson noise, (c) Deconvolved result using ℓ_2-optimization assuming Gaussian noise, and (d) Reconstruction using our optimization assuming Poisson noise. This example demonstrates that proper noise modeling leads to significant gains in reconstruction quality for large blur kernels. To only demonstrate the effect of the noise model, we did* not use cross-channel information *for this example. Since the Poisson distribution can be approximated well by a normal distribution for large values of the mean, the most significant improvements of using the proper noise model can be found in the low-intensity regions.*

$36.45dB$ for [Heide et al. 2013] and $36.44dB$ for our approach. The method from [Yue et al. 2015] suffers from inaccuracy for large values of β and therefore converges slowly as shown in Supplementary Fig. 5. The algorithm parameters of the methods we compare to have been tuned for best performance.

Parameters The algorithm parameters have been adopted from [Krishnan and Fergus 2009]. Setting $\alpha = 2 \cdot \sqrt{2}$ and 5 iterations was sufficient for all simulation and real-world results from our work. The parameters of the objective μ, γ do in general depend on the standard deviation of the noise and the blur kernel [Heide et al. 2014]. However, the cross-channel prior makes our method fairly robust to the parameter selection. We use $\mu = 1$ and select γ for a given lens prototype. Note that all parameter values are fixed for a given lens prototype and only one parameter needs to be selected in our method.

3.2.5 Poisson Noise Fitting

We can also easily modify our method to optimize for observations degraded by Poisson noise. In this case, we model the observed image $\mathbf{j}$ as a sample of a random variable $\tilde{\mathbf{j}}$:

$$p(\tilde{\mathbf{j}} = \mathbf{j} \mid \lambda) = \prod_{i=1}^{n} \frac{\lambda_i^{\mathbf{j}_i} e^{-\lambda_i}}{\mathbf{j}_i!}, \tag{22}$$

where here the notation $(\cdot)_i$ denotes the selection of the i-th component of the image vector given as argument. Following the Bayesian maximum a posteriori criterion, which is also proposed in [Figueiredo and Bioucas-Dias 2009], the quadratic data-term $\|\mathbf{X}\mathbf{v} - \mathbf{j}\|_2^2$ from Eq. (15) then becomes the negative log-likelihood of $p(\tilde{\mathbf{j}} = \mathbf{j} \mid \mathbf{v})$, that is

$$\begin{aligned} -\log\left(p(\tilde{\mathbf{j}} = \mathbf{j} \mid \mathbf{v})\right) &= \sum_{i=1}^{n} \Gamma\left((\mathbf{X}\mathbf{v})_i, \mathbf{j}_i\right) \quad \text{with} \\ \Gamma(a,b) &= a - b\log(a) + \mathrm{ind}_{\mathbb{R}^+}(a), \end{aligned} \tag{23}$$

where $\mathrm{ind}_{\mathbb{R}^+}(a)$ is the indicator function for the positive orthant. We can now easily bring this into our generalized objective form from Eq. (17) by setting

$$f_1(\mathbf{p}) = \mathbf{p} - \mathbf{j}\log(\mathbf{p}) + \mathrm{ind}_{\mathbb{R}^+}(\mathbf{p}) \tag{24}$$

The corresponding proximal operator for the changed f_1 is

$$\mathbf{prox}_{\theta f_1(\cdot)}(\mathbf{p}) = \frac{\mathbf{p} - \theta}{2} + \sqrt{\theta\mathbf{j} + \frac{(\theta - \mathbf{p})^2}{4}} \quad \textbf{Poisson Penalty} \tag{25}$$

This analytic solution for the proximal operator of f_1 results in a root-finding problem of a second-order polynomial as shown in Eq. (26). Due to the positivity constraint in f_1 the minimum is uniquely defined by the positive root:

$$\begin{aligned} \mathbf{prox}_{\theta f_1(\cdot)}(\mathbf{p}) &= \underset{\mathbf{p}}{\operatorname{argmin}} \ f_1(\mathbf{p}) + \frac{1}{2\theta}\|\mathbf{p} - \mathbf{p}\|_2^2 \\ &= \underset{\mathbf{p} \in \mathbb{R}^+}{\operatorname{argmin}} \ \underbrace{\mathbf{p} - \mathbf{j}\log(\mathbf{p}) + \frac{1}{2\theta}\|\mathbf{p} - \mathbf{p}\|_2^2}_{\Upsilon(\mathbf{p})} \\ \Leftrightarrow \frac{\partial \Upsilon(\mathbf{p}_{\text{opt}})}{\partial \mathbf{p}} &= \mathbf{1} - \frac{\mathbf{j}}{\mathbf{p}_{\text{opt}}} + \frac{1}{\theta}\mathbf{p}_{\text{opt}} - \frac{1}{\theta}\mathbf{p} \overset{!}{=} 0 \quad \text{s.t.} \quad \mathbf{p}_{\text{opt}} \in \mathbb{R}^+ \\ \Leftrightarrow \mathbf{p}_{\text{opt}}^2 + (\theta - \mathbf{p}) \cdot \mathbf{p}_{\text{opt}} - \theta\mathbf{j} &= 0 \quad \text{s.t.} \quad \mathbf{p}_{\text{opt}} \in \mathbb{R}^+ \\ \Leftrightarrow \mathbf{p}_{\text{opt}} &= \frac{\mathbf{p} - \theta}{2} + \sqrt{\theta\mathbf{j} + \frac{(\theta - \mathbf{p})^2}{4}} \end{aligned}$$

Having defined the changed f_1 and the corresponding proximal operator, we can directly apply our framework derived in Sec 3.2.2, which maps directly to the ADMM algorithm or Half-Quadratic Splitting method as described above. The only component that changes is that the set Ψ is now empty (since f_1 is now no longer a simple quadratic as defined previously) and consequently $\Psi := \{1, 2, 3\}$. This means new auxiliary variables for $\mathbf{K}_i\mathbf{v}$ are introduced. The price, that we pay for being able to solve for Poisson degraded observations are now again more iterations (necessary to enforce the consensus constraints). However, the quality is quite significantly affected by the more proper noise model as demonstrated in Supplementary Fig. 6. Note also that we cannot simply use a variance stabilization transform here (such as the Anscombe transform [Mäkitalo and Foi 2011]) since the observations are mixed together in the convolution with the kernel.

4 Fabrication

Each encodable lens consists of two phase-only multi-level DOEs. They are fabricated on Fused Silica substrates by four iterations of photolithography and Reactive Ion Etching (RIE) techniques.

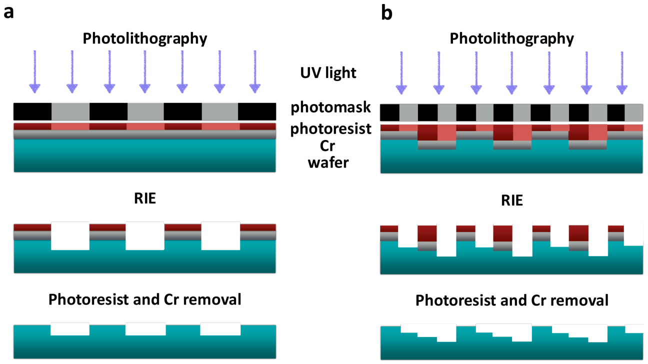

Supplementary Figure 7: Fabrication of multi-level DOEs. *Each iteration creates 2-level microstructures on the previous profile by applying photolithography followed by RIE and removal of auxiliary layers. By repeating the fabrication cycle, one can obtain multi-level microstructures on the substrate.*

Supplementary Figure 7 shows the the iterative process of photolithography and RIE to fabricate multi-level DOEs. In each iteration, a thin Chromium (Cr) layer is first deposited on the substrate wafer. A photoresist layer is then spin-coated on the Cr layer and gains its shape after a softbake process. The designed patterns are transferred from the photomask to the photoresist under UV light exposure with a certain dose. After exposure, the chemical property of the exposed area on the photoresist changes and can consequently be removed in the developer in a well controlled development environment. Subsequently, the open area of the Cr layer is removed in Cr etchant and the patterns are transferred to the substrate. In the RIE step, the material in the open area is removed by plasma and a certain height profile is created on the substrate after removing the auxiliary layers. Each fabrication cycle doubles the number of microstructure levels on the previous profile. Repeating this cycle by 4 iterations, we can obtain 16 levels of microstructure on the substrate.

Fabrication details are as follows.

Wafer. We choose 4 inch fused silica wafers with $0.5mm$ thickness as the substrate. Fused silica has high transmittance in the entire visible band. It also has a low coefficient of thermal expansion ($5.5 \times 10^{-7}/°C$) and pure chemical composition ($> 99.999\%$ SiO_2), which makes it a good candidate for both photolithpgraphy and RIE.

Photomask. Our mask patterns were created on a 5 inch Soda Lime photomask using direct laser writing. A blank photomask is a substrate with precoated Cr and photoresist. The photoresist is exposed in the laser direct writer Heidelberg DWL2000. To fabricate 16-level DOEs, 4 masks are needed. We designed an array of 8000 $\times$ 8000 pixels for each mask and the size of each pixel is $1\mu m$.

Wafer Preprocessing. Before photolithography, blank fused silica wafers are cleaned in 120 °C Piranha solution (mixture of H_2SO_4 and H_2O_2 3:1) for 20 min. Metals and organic contamination are removed during this process. Wafers are then rinsed in deionized (DI) water for 2 min followed by N_2 drying for 7 min. A thin Cr film ($200nm$) is deposited on one side of the cleaned wafers by sputter deposition in an ESC Reactive and Metal Sputter System. This auxiliary layer helps making the surface reflective instead of transmissive.

Photolithography. The photolithography process consists of three major steps: spin coating, UV exposure and development. In order to promote the photoresist-to-wafer adhesion, the wafers are treated in a vapor prime step with Hexamethyldisilazane (HMDS) first. A positive photoresist AZ1505 is then spin coated on the wafer followed by a 60 sec softbake to form a $0.6\mu m$ layer. In the UV exposure step, the wafer is aligned with the photomask on a mask aligner EVG 6200∞. The maximum alignment resolution is $1\mu m$ in a Vacuum + Hard contact mode. A constant dose of $15mJ/cm^2$ UV exposure is applied to the wafer. In development, MIF AZ726 (2.38% TMAH in H_2O) serves as the developer. A 20 sec development followed by DI water rinse removes the photoresist in the exposed areas.

RIE. After development, patterns on the photoresist have to be transferred to the Cr layer before RIE. This step is done in Cr etchant (mixtures of $HClO_4$ and $NH_4)_2[Ce(NO_3)_6]$ until all the open areas on the Cr layer are removed. Subsequently, the residual photoresist is removed with Aceton in an ultrasonic treatment for 5 min. The patterned Cr severs as a hard mask in RIE. Fused silica etching is done in a vacuum chamber in Oxford PlasmaLab System 100 with mixed gases of Argon and SF_6 (8:2). The etching rate is $90nm/min$ for fused silica. For our 16-level DOEs, each level corresponds to a depth of $75nm$ for design wavelength $\lambda_0 = 550nm$. Therefore the etching durations are 0.83 min, 1.66 min, 3.32 min and 6.64 min respectively. To keep the aspect ratio as high as possible and also stabilize the process, the RIE works in a etch-idle-etch loop mode.

5 Applications

We show more results for static broadband imaging and zooming using our encodable diffractive lenses.

5.1 Static Broadband Imaging

See the results in Supplementary Figs. 8, 10, 11 and12.

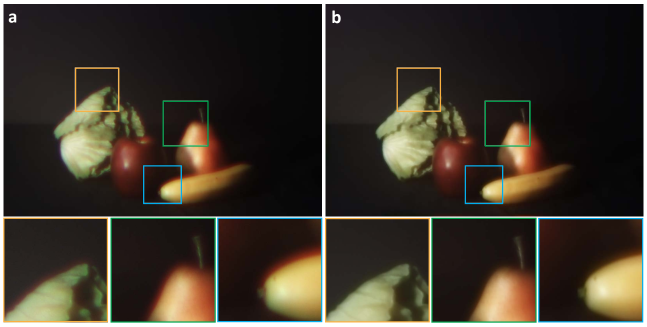

Supplementary Figure 8: Images captured with a single encodable lens (single diffractive interface). *A set of representable scenes under indoor lighting conditions is shown here. We compare the aberrated capture (a) and restored with our method (b). Small colour insets below each image highlight details and illustrate how our method removes the large severely wavelength dependent aberrations.*

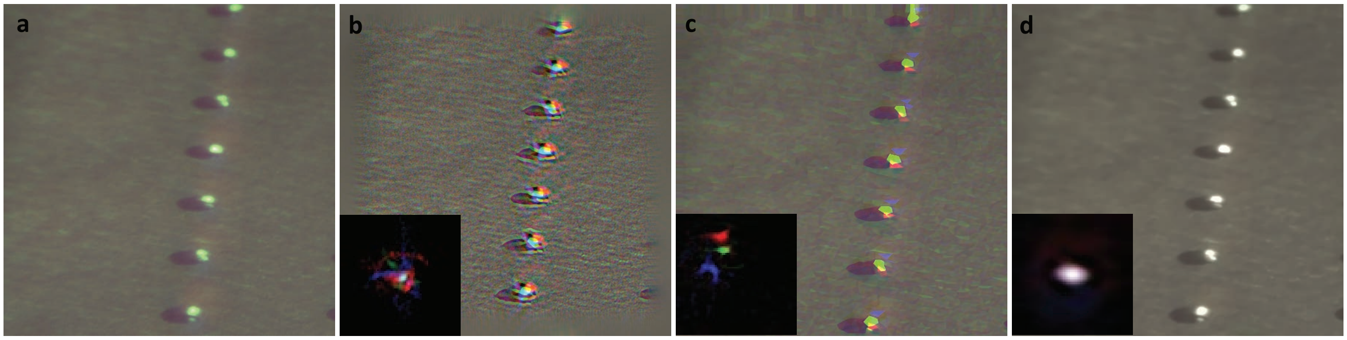

Blurred observation

Method from [Sun et al. 2013] **(Runtime: 0 hours, 4 min, 19 sec)**

Method from [Michaeli and Irani 2014] **(Runtime 16 hours, 36 min, 53 sec)**

Using our cross-channel method **(Runtime 0 hours, 0 min, 6 sec)**

Supplementary Figure 9: Comparison of our blind PSF estimation method against the state-of-the-art. *We have extracted a patch of a blurry observation (left image) and compare the PSF estimate and latent corrected image of state-of-the-art deconvolution methods versus our method. We can see that the methods of [Sun et al. 2013] and [Michaeli and Irani 2014] actually diverge in this case and increase chromatic aberrations rather than removing them. Since the PSF is very large for this patch (around* 100×100 *pixels), most competing methods are very expensive. In fact [Michaeli and Irani 2014] took* more than 16 hours *for the computation of this patch.*

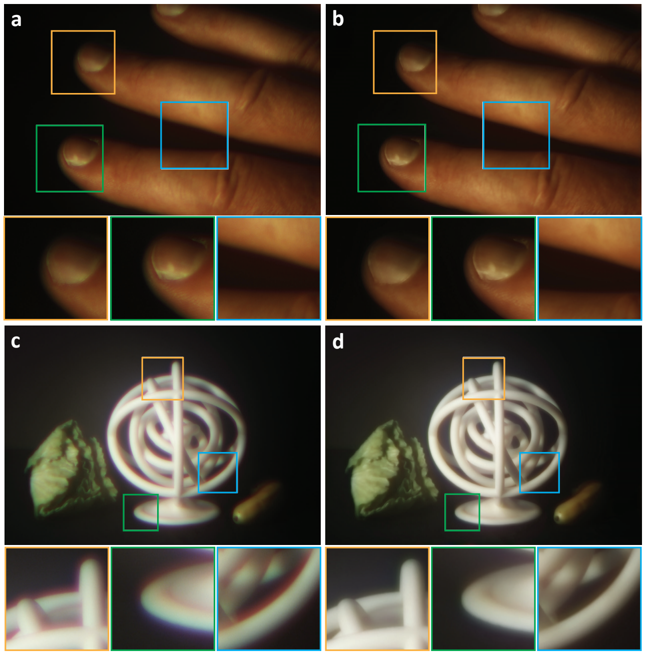

Supplementary Figure 10: Images captured with a single encodable lens (single diffractive interface). *In addition to still lives from Supplementary Fig. 8 we show here also refractive objects and skin. We compare the aberrated capture (a,c) and restored with our method (b,d). Small colour insets below each image highlight details and illustrate how our method removes the large severely wavelength dependent aberrations.*

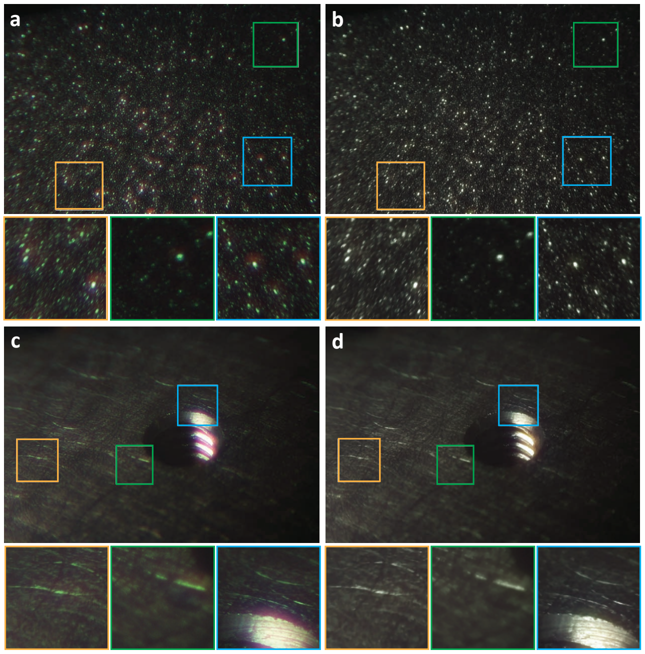

Supplementary Figure 11: Extremely short focus setting (macro) capture. *Images are captured with the same single encodable lens that was used for the captures in Supplementary Figs. 8 and 10. Again, we compare here the aberrated capture (a,c) and restored with our method (b,d). Small colour insets below each image highlight details.*

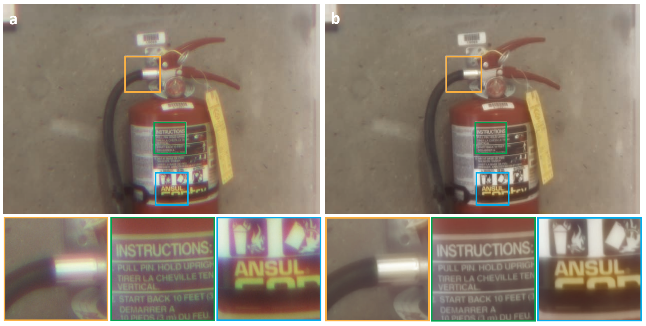

Supplementary Figure 12: Outdoor scene. *Images are captured with the same single encodable lens that was used in Supplementary Figs. 8, 10 and 11. Again, we compare here the aberrated capture (a) and restored with our method (b). Small colour insets below each image highlight details.*

5.2 Mixed-focus Zooming

When two focus settings are blended at the same time, images with different zooming ratios overlap on the same image. See Supplementary Fig. 13.

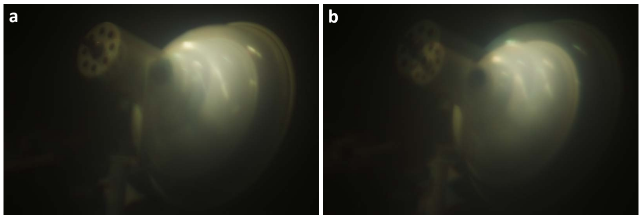

Supplementary Figure 13: Mixed lens encodings. *Lens encoding where we blend between two different focus levels resulting in a mixture of two different zoom settings. This figure also demonstrates the relative zoom ratio we can achieve.*

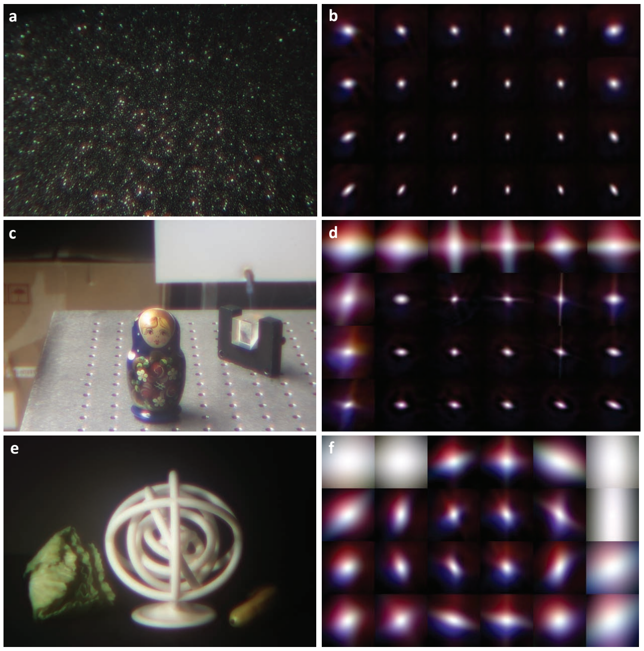

Supplementary Figure 14: Different PSFs estimated during our blind calibration procedure. *We show here the aberrated measurement (left column) and the corresponding estimated PSFs (right column). We can see that for all images our method calibrates a very large PSF with a peak and large, low-frequency low red and blue components. Especially in the closeup form image we can see that the measured PSF is a reasonable guess since the locations around the peaky highlights show the shape of the local PSF in this case. For completely flat image regions with no features to learn from, our method can only use the smoothness constraint (hence the large blurs, which is a measure of uncertainty in this case). Note that the flat regions do not cause issues in the reconstruction.*

6 Evaluation

6.1 PSF Estimation of Measured Data

See the results in Supplementary Fig. 14 and for a comparison to the state-of-the-art in Supplementary Fig. 9.

6.2 PSF Estimation in Simulation

For all of our comparisons in simulation we use the KODAK dataset [Kodak 2015].

Supplementary Table. 2 shows simulation results for our blind PSF estimation.

6.3 Non-Blind Deconvolution in Simulation

Supplementary Table. 3 shows simulation results for the non-blind deconvolution part of our method evaluated on its own.

6.4 Performance

Supplementary Fig. 15 shows runtime values for the blind estimation methods averaged over all test cases from Supplementary Table. 2.

Case (a)

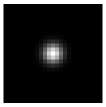

Method	1	2	3	4	5	6	7	8	9	10	11	12	13	14	15	Avg
Sun et. al. [2013]	21.14	22.52	25.67	24.55	31.31	27.06	29.01	21.31	26.06	23.65	28.50	28.85	26.22	28.85	28.87	26.24
Schuler et al. [2012]	17.90	18.49	17.95	19.70	16.99	18.57	25.19	18.82	18.03	18.74	17.89	21.22	17.66	18.29	19.88	19.02
Michaeli and Irani [2014]	15.92	17.35	16.88	17.35	15.41	16.57	16.23	15.39	16.14	17.62	15.93	16.60	14.88	15.58	16.71	16.31
Ours	**30.71**	**29.71**	**29.99**	**29.82**	**35.40**	**31.03**	**32.02**	**32.69**	**32.67**	**30.20**	**31.83**	**32.50**	**32.17**	**33.68**	**30.77**	**31.68**

Case (b)

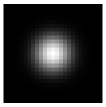

Method	1	2	3	4	5	6	7	8	9	10	11	12	13	14	15	Avg
Sun et. al. [2013]	18.22	17.12	19.04	18.73	24.53	15.39	24.78	22.06	19.53	17.82	23.90	22.68	20.33	23.97	21.82	20.66
Schuler et al. [2012]	12.38	13.31	12.37	18.13	10.86	13.52	11.74	13.19	21.12	13.10	12.11	12.91	11.52	13.36	18.45	13.87
Michaeli and Irani [2014]	11.78	14.39	12.69	15.00	11.64	14.00	11.57	11.00	12.91	14.79	11.95	13.14	10.87	11.04	12.45	12.61
Ours	**27.98**	**30.74**	**26.54**	**28.73**	**30.69**	**30.00**	**29.56**	**31.95**	**35.21**	**30.15**	**30.54**	**33.00**	**28.48**	**30.79**	**31.25**	**30.37**

Case (c)

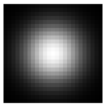

Method	1	2	3	4	5	6	7	8	9	10	11	12	13	14	15	Avg
Sun et. al. [2013]	7.87	7.97	8.44	7.99	11.09	7.89	9.71	11.26	8.26	7.19	9.12	9.37	8.86	10.00	8.81	8.92
Schuler et al. [2012]	10.85	12.09	12.26	12.13	11.51	12.69	12.08	9.56	11.83	12.73	12.35	10.47	10.48	11.33	13.74	11.74
Michaeli and Irani [2014]	9.85	12.59	10.45	14.43	9.32	12.19	8.85	8.98	10.95	13.67	10.79	11.15	9.42	9.17	9.77	10.77
Ours	**19.84**	**24.81**	**18.88**	**19.47**	**21.24**	**22.35**	**22.66**	**24.68**	**24.44**	**21.92**	**22.94**	**23.60**	**22.30**	**22.00**	**25.78**	**22.46**

Case (d)

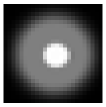

Method	1	2	3	4	5	6	7	8	9	10	11	12	13	14	15	Avg
Sun et. al. [2013]	6.40	7.19	7.39	6.83	9.53	1.41	8.24	9.42	7.07	6.70	7.81	8.12	7.87	8.56	7.35	7.33
Schuler et al. [2012]	12.68	12.88	12.80	12.72	13.75	13.58	13.43	12.05	13.13	14.23	12.56	12.68	11.77	12.10	14.65	13.00
Michaeli and Irani [2014]	9.66	12.69	10.39	13.53	9.35	11.63	8.77	9.34	11.12	12.73	10.72	10.51	9.70	9.23	8.86	10.55
Ours	**18.18**	**23.35**	**18.05**	**17.71**	**20.30**	**21.20**	**22.18**	**23.52**	**22.96**	**19.54**	**22.04**	**22.15**	**21.71**	**20.81**	**24.18**	**21.19**

Case (d)

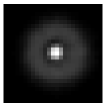

Method	1	2	3	4	5	6	7	8	9	10	11	12	13	14	15	Avg
Sun et. al. [2013]	18.83	19.30	19.18	19.23	21.20	19.19	20.57	21.22	19.35	19.01	19.74	19.86	19.40	20.79	19.66	19.77
Schuler et al. [2012]	19.88	20.61	20.60	20.74	20.02	20.63	20.00	19.04	20.92	21.08	20.35	19.67	19.20	19.82	22.33	20.33
Michaeli and Irani [2014]	18.53	20.62	19.92	21.43	19.02	20.65	18.97	18.64	19.67	20.95	19.66	20.07	18.62	19.10	19.61	19.70
Ours	**28.64**	**30.87**	**29.49**	**29.51**	**32.95**	**31.86**	**31.46**	**34.73**	**33.15**	**31.61**	**32.93**	**32.78**	**33.32**	**32.55**	**31.82**	**31.84**

Case (e)

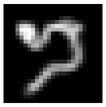

Method	1	2	3	4	5	6	7	8	9	10	11	12	13	14	15	Avg
Sun et. al. [2013]	8.87	10.08	10.92	10.23	10.17	10.74	11.16	10.97	10.19	10.01	10.31	11.46	9.69	10.37	12.08	10.48
Schuler et al. [2012]	10.75	13.76	11.73	12.01	10.56	11.44	11.01	10.47	11.25	12.37	11.26	11.31	10.52	11.04	13.16	11.51
Michaeli and Irani [2014]	9.15	11.38	11.42	11.11	8.92	10.37	8.95	9.19	9.78	8.59	10.43	9.76	8.49	10.10	9.67	9.82
Ours	**23.35**	**22.07**	**22.41**	**20.91**	**27.71**	**23.84**	**23.31**	**25.99**	**24.81**	**22.62**	**24.84**	**24.55**	**25.94**	**25.81**	**23.29**	**24.10**

Supplementary Table 2: Comparison of our *blind estimation method* to four other state-of-the-art methods. *PSNR for the PSF (in dB) is given for each image in the dataset. We consider 6 different representative PSFs, shown to the left of each individual PSNR table. PSNR averages for all images are shown in the very last column of each table.*

Case (a)

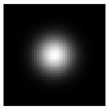

Method	1	2	3	4	5	6	7	8	9	10	11	12	13	14	15	Avg
Blurred	22.43	30.21	29.61	26.40	20.95	24.98	24.72	19.38	25.27	26.53	25.24	27.58	21.64	24.30	26.87	25.08
Yue et. al. [2015]	23.58	31.48	31.67	28.45	22.55	26.05	26.50	20.76	26.85	28.42	26.75	29.72	22.58	25.86	29.61	26.72
Schuler et al. [2011]	31.72	36.14	35.74	31.67	30.97	33.14	32.92	29.53	34.02	34.66	34.50	33.97	31.02	31.43	34.14	33.04
Ours	**36.05**	**36.35**	**38.98**	**33.14**	**33.40**	**38.39**	**36.31**	**33.51**	**36.82**	**37.26**	**36.98**	**39.02**	**35.23**	**31.13**	**36.08**	**35.91**

Case (b)

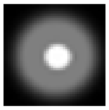

Method	1	2	3	4	5	6	7	8	9	10	11	12	13	14	15	Avg
Blurred	21.20	28.79	27.29	24.10	19.29	23.62	22.97	17.94	23.64	24.49	23.59	25.26	20.59	22.60	24.02	23.29
Yue et. al. [2015]	22.40	30.27	29.63	26.37	20.83	24.94	24.76	19.24	25.29	26.51	25.18	27.50	21.62	24.23	26.99	25.05
Schuler et al. [2011]	28.80	33.38	31.82	27.65	27.59	28.98	30.04	26.49	30.92	31.02	31.67	30.50	27.54	28.84	29.99	29.68
Ours	**34.04**	**34.14**	**36.20**	**31.45**	**30.89**	**36.73**	**34.33**	**32.01**	**34.94**	**35.45**	**34.69**	**36.52**	**33.98**	**29.26**	**34.30**	**33.93**

Case (c)

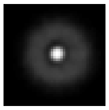

Method	1	2	3	4	5	6	7	8	9	10	11	12	13	14	15	Avg
Blurred	21.85	29.51	28.25	25.08	20.07	24.37	23.84	18.63	24.43	25.42	24.38	26.25	21.20	23.42	25.13	24.12
Yue et. al. [2015]	23.52	31.35	31.01	27.91	22.11	26.01	26.35	20.46	26.53	27.91	26.43	29.00	22.57	25.54	28.63	26.36
Schuler et al. [2011]	31.29	34.01	33.11	31.01	30.16	30.93	32.09	29.02	32.49	32.44	32.64	32.01	30.11	31.27	32.10	31.65
Ours	**36.56**	**36.69**	**39.03**	**34.42**	**34.03**	**38.26**	**37.29**	**34.34**	**38.19**	**37.71**	**37.08**	**38.99**	**35.86**	**33.42**	**36.98**	**36.59**

Case (d)

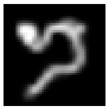

Method	1	2	3	4	5	6	7	8	9	10	11	12	13	14	15	Avg
Blurred	20.39	28.00	26.44	23.28	18.60	22.57	22.07	17.10	22.83	23.53	22.69	24.15	19.78	21.72	23.32	22.43
Yue et. al. [2015]	24.03	31.84	31.72	28.71	22.84	26.49	27.22	21.12	27.07	28.63	27.02	29.90	22.97	26.23	29.54	27.02
Schuler et al. [2011]	28.42	33.65	31.64	26.60	27.44	28.21	28.80	24.79	29.36	28.73	30.65	29.10	27.06	28.56	27.72	28.71
Ours	**37.89**	**38.00**	**40.23**	**35.11**	**35.54**	**39.18**	**38.80**	**35.01**	**38.54**	**38.52**	**38.24**	**40.39**	**36.40**	**34.53**	**37.66**	**37.60**

Case (e)

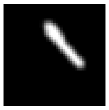

Method	1	2	3	4	5	6	7	8	9	10	11	12	13	14	15	Avg
Blurred	21.17	28.17	26.26	23.29	18.26	23.07	22.53	17.98	23.26	24.21	23.15	24.65	20.04	21.87	23.27	22.74
Yue et. al. [2015]	24.35	32.36	32.68	29.73	23.74	26.64	27.76	21.57	27.67	29.11	27.52	30.69	23.24	26.78	30.71	27.64
Schuler et al. [2011]	29.77	33.45	32.66	30.04	29.15	28.74	30.94	27.36	31.50	31.87	32.61	30.93	27.82	30.25	30.91	30.53
Ours	**38.40**	**38.61**	**40.80**	**36.06**	**36.44**	**39.27**	**38.83**	**35.87**	**39.25**	**39.03**	**38.71**	**40.77**	**36.82**	**35.19**	**38.30**	**38.16**

Supplementary Table 3: Comparison of our *non-blind cross-channel deconvolution method* to four other state-of-the-art methods. *PSNR (in dB) is given for each image in the dataset. We consider 5 different representative PSFs, shown to the left of each individual PSNR table. PSNR averages for all images are shown in the very last column of each table.*

Supplementary Fig. 16 shows the runtime values for the non-blind deconvolution methods averaged over all test cases from Supplementary Table. 3.

6.5 MTF Analysis

The goal of our method is to provide the design flexibility for defining the transmission function of a DOE. Compared with the analytical method investigated in [Bernet and Ritsch-Marte 2008], our optimization gives competitive results. To the functionality of adjustable focal power, Supplementary Fig.17

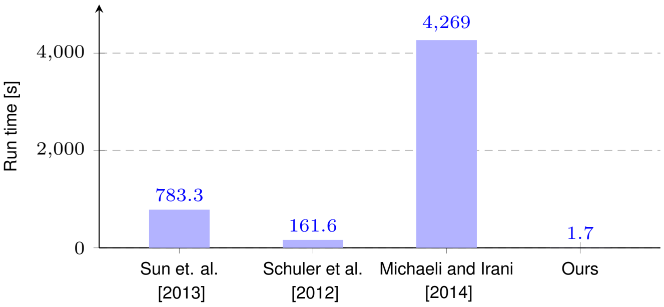

Supplementary Figure 15: Performance in run time of blind PSF estimation on synthetic data (lower is better). *This plot shows the average run time of all experiments from Supplementary Table. 2.*

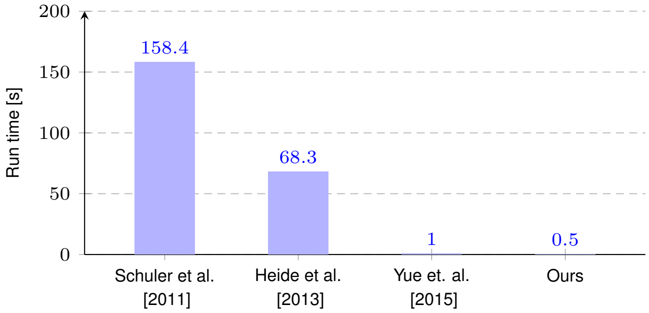

Supplementary Figure 16: Performance in run time of blind PSF estimation on synthetic data (lower is better). *This plot shows the average run time of all experiments from Supplementary Table. 3.*

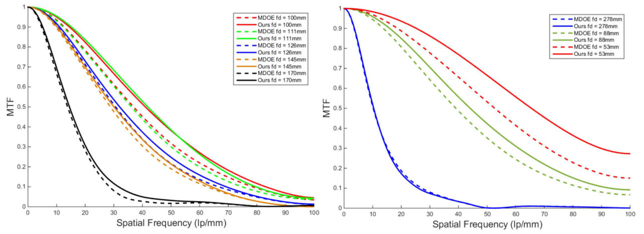

Supplementary Figure 17: MTF comparisons on our optimized diffractive lens (solid graphs) and corresponding MDOE design (dash graphs). *Different colours indicate the MTFs at different focal lengths. The designed wavelength is set* $\lambda = 550nm$.

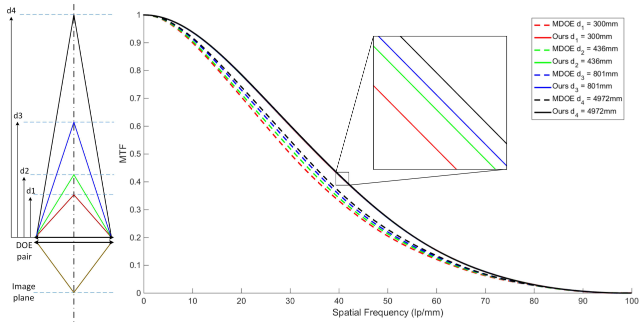

Supplementary Figure 18: Refocusing comparisons on our optimized diffractive lens (solid graphs) and corresponding MDOE design (dash graphs). *Different colours indicate the lens focusing at different object distances. The designed wavelength is set* $\lambda = 550nm$.

shows respectively the Modulation Transfer Function (MTF) curves of our optimized diffractive lens and a MDOE design [Bernet and Ritsch-Marte 2008; Bernet et al. 2013] at the same wavelength while different focal lengths, subject to tune different relative rotation angles. The focal length ranges are $[100mm, 200mm]$ (left sub-figure) and $(-\infty, -50mm] \cup [50mm, \infty)$ (right sub-figure), respectively. The MTF curves indicated are thus readily obtained by first acquiring the image of an incoherent point source (synthetic PSFs), and applying the two-dimensional discrete Fourier transform to the sampled PSF distribution. We observe that MTFs of our designs (solid graphs) present averagely better performance than that of MDOE designs (dash graphs) at different focal lengths, indicated as different colours. Note that all curves with larger focal lengths drop more significantly than that of shorter ones, which is consistent with the fact that theoretical cut-off frequency is in inverse proportion to F number.

Supplementary Fig. 18 shows the MTF comparisons of our optimized encoded lens and a MDOE design with the same focal length range $[100mm, 200mm]$. Both are fixed at the same image plane while focusing at different object distances. Our factorization based design indeed has achieved a competitive compromise within the adjustable focal range.

Supplementary References

ALMEIDA, M. S., AND FIGUEIREDO, M. A. 2013. Frame-based image deblurring with unknown boundary conditions using the alternating direction method of multipliers. In *Proc. ICIP*, 582–585.

BERNET, S., AND RITSCH-MARTE, M. 2008. Adjustable refractive power from diffractive moiré elements. *Appl. Opt. 47*, 21, 3722–3730.

BERNET, S., HARM, W., AND RITSCH-MARTE, M. 2013. Demonstration of focus-tunable diffractive moiré-lenses. *Opt. Express 21*, 6, 6955–6966.

BOYD, S., PARIKH, N., CHU, E., PELEATO, B., AND ECKSTEIN, J. 2011. Distributed optimization and statistical learning via the alternating direction method of multipliers. *Foundations and Trends in Machine Learning 3*, 1, 1–122.

CHAMBOLLE, A., AND POCK, T. 2011. A first-order primal-dual algorithm for convex problems with applications to imaging. *Journal of Mathematical Imaging and Vision 40*, 1, 120–145.

DENG, J., DONG, W., SOCHER, R., LI, L.-J., LI, K., AND FEI-FEI, L. 2009. Imagenet: A large-scale hierarchical image database. In *Proc. IEEE CVPR*, IEEE, 248–255.

FIGUEIREDO, M. A., AND BIOUCAS-DIAS, J. M. 2009. Deconvolution of poissonian images using variable splitting and augmented lagrangian optimization. In *IEEE Statistical Signal Processing*, IEEE, 733–736.

GEMAN, D., AND YANG, C. 1995. Nonlinear image recovery with half-quadratic regularization. *IEEE TIP 4*, 7, 932–946.

GOODMAN, J. W. 2005. *Introduction to Fourier optics*. Roberts and Company Publishers.

HAEFFELE, B., YOUNG, E., AND VIDAL, R. 2014. Structured low-rank matrix factorization: Optimality, algorithm, and applications to image processing. In *Proc. ICML*, 2007–2015.

HEIDE, F., ROUF, M., HULLIN, M. B., LABITZKE, B., HEIDRICH, W., AND KOLB, A. 2013. High-quality computational imaging through simple lenses. *ACM Trans. Graph. 32*, 5, 149.

HEIDE, F., STEINBERGER, M., TSAI, Y.-T., ROUF, M., PAJĄK, D., REDDY, D., GALLO, O., LIU, J., HEIDRICH, W., EGIAZARIAN, K., ET AL. 2014. Flexisp: a flexible camera image processing framework. *ACM Transactions on Graphics (TOG) 33*, 6, 231.

HEIDE, F., HEIDRICH, W., AND WETZSTEIN, G. 2015. Fast and flexible convolutional sparse coding. In *Proc. IEEE CVPR*, IEEE, 5135–5143.

HO, N.-D. 2008. *Nonnegative matrix factorization algorithms and applications*. PhD thesis, ÉCOLE POLYTECHNIQUE.

KODAK, 2015. Kodak lossless true color image suite.

KRISHNAN, D., AND FERGUS, R. 2009. Fast image deconvolution using hyper-laplacian priors. In *Advances in Neural Information Processing Systems*, 1033–1041.

LAI, W.-S., DING, J.-J., LIN, Y.-Y., AND CHUANG, Y.-Y. 2015. Blur kernel estimation using normalized color-line priors. In *Proc. IEEE CVPR*, IEEE, 64–72.

MÄKITALO, M., AND FOI, A. 2011. Optimal inversion of the anscombe transformation in low-count poisson image denoising. *IEEE TIP 20*, 1, 99–109.

MARKOVSKY, I. 2011. Algorithms and literate programs for weighted low-rank approximation with missing data. In *Approximation algorithms for complex systems*. Springer, 255–273.

MICHAELI, T., AND IRANI, M. 2014. Blind deblurring using internal patch recurrence. In *IEEE ECCV*. Springer, 783–798.

PERRONE, D., AND FAVARO, P. 2014. Total variation blind deconvolution: The devil is in the details. In *Proc. IEEE CVPR*, IEEE.

RUSSELL, B. C., TORRALBA, A., MURPHY, K. P., AND FREEMAN, W. T. 2008. Labelme: a database and web-based tool for image annotation. *International journal of computer vision 77*, 1-3, 157–173.

SCHULER, C. J., HIRSCH, M., HARMELING, S., AND SCHOLKOPF, B. 2011. Non-stationary correction of optical aberrations. In *Proc. IEEE ICCV*, IEEE, 659–666.

SCHULER, C. J., HIRSCH, M., HARMELING, S., AND SCHÖLKOPF, B. 2012. Blind correction of optical aberrations. In *IEEE ECCV*. Springer, 187–200.

SUN, L., CHO, S., WANG, J., AND HAYS, J. 2013. Edge-based blur kernel estimation using patch priors. In *Proc. IEEE ICCP*, IEEE, 1–8.

XU, Y., AND YIN, W. 2013. A block coordinate descent method for regularized multiconvex optimization with applications to nonnegative tensor factorization and completion. *SIAM Journal on imaging sciences 6*, 3, 1758–1789.

YUE, T., SUO, J., WANG, J., CAO, X., AND DAI, Q. 2015. Blind optical aberration correction by exploring geometric and visual priors. In *Proc. IEEE CVPR*, IEEE, 1684–1692.
